# SARS-CoV-2 Orf6 hijacks Nup98 to block STAT nuclear import and antagonize interferon signaling

**DOI:** 10.1073/pnas.2016650117

**Published:** 2020-10-23

**Authors:** Lisa Miorin, Thomas Kehrer, Maria Teresa Sanchez-Aparicio, Ke Zhang, Phillip Cohen, Roosheel S. Patel, Anastasija Cupic, Tadashi Makio, Menghan Mei, Elena Moreno, Oded Danziger, Kris M. White, Raveen Rathnasinghe, Melissa Uccellini, Shengyan Gao, Teresa Aydillo, Ignacio Mena, Xin Yin, Laura Martin-Sancho, Nevan J. Krogan, Sumit K. Chanda, Michael Schotsaert, Richard W. Wozniak, Yi Ren, Brad R. Rosenberg, Beatriz M. A. Fontoura, Adolfo García-Sastre

**Affiliations:** ^a^Department of Microbiology, Icahn School of Medicine at Mount Sinai, New York, NY 10029;; ^b^Global Health Emerging Pathogens Institute, Icahn School of Medicine at Mount Sinai, New York, NY 10029;; ^c^Department of Cell Biology, University of Texas Southwestern Medical Center, Dallas, TX 75390;; ^d^Department of Cell Biology, University of Alberta, Edmonton, AB T6G 2H7, Canada;; ^e^Department of Biochemistry, Vanderbilt University School of Medicine, Nashville, TN 37232;; ^f^Immunity and Pathogenesis Program, Infectious and Inflammatory Disease Center, Sanford Burnham Prebys Medical Discovery Institute, La Jolla, CA 92037;; ^g^Quantitative Biosciences Institute, University of California San Francisco, CA 94158;; ^h^Gladstone Institute of Data Science and Biosciences, J. David Gladstone Institutes, San Francisco, CA 94158;; ^i^Department of Cellular and Molecular Pharmacology, University of California San Francisco, CA 94143;; ^j^Department of Medicine, Division of Infectious Diseases, Icahn School of Medicine at Mount Sinai, New York, NY 10029;; ^k^Tisch Cancer Institute, Icahn School of Medicine at Mount Sinai, New York, NY 10029

**Keywords:** SARS-CoV-2, interferon signaling antagonism, STATs, ORF6, Nup98

## Abstract

Severe acute respiratory syndrome coronavirus 2 (SARS-CoV-2) is the causative agent of the ongoing coronavirus disease 2019 (COVID-19) pandemic that is a serious global health problem. Evasion of IFN-mediated antiviral signaling is a common defense strategy that pathogenic viruses use to replicate and propagate in their host. In this study, we show that SARS-CoV-2 is able to efficiently block STAT1 and STAT2 nuclear translocation in order to impair transcriptional induction of IFN-stimulated genes (ISGs). Our results demonstrate that the viral accessory protein Orf6 exerts this anti-IFN activity. We found that SARS-CoV-2 Orf6 localizes at the nuclear pore complex (NPC) and directly interacts with Nup98-Rae1 via its C-terminal domain to impair docking of cargo-receptor (karyopherin/importin) complex and disrupt nuclear import. In addition, we show that a methionine-to-arginine substitution at residue 58 impairs Orf6 binding to the Nup98-Rae1 complex and abolishes its IFN antagonistic function. All together our data unravel a mechanism of viral antagonism in which a virus hijacks the Nup98-Rae1 complex to overcome the antiviral action of IFN.

## Significance

To successfully establish infection, viral pathogens have to overcome the interferon (IFN)-mediated antiviral response. Previous studies revealed that the viral accessory protein Orf6 of SARS-CoV and SARS-CoV-2 is able to inhibit STAT1 nuclear translocation to block IFN signaling. In this study, we report that Orf6 localizes at the nuclear pore complex (NPC) where it binds directly to the Nup98-Rae1 complex to target the nuclear import pathway and mediate this inhibition. A better understanding of the strategies used by viruses to subvert host immune responses is critical for the design of novel antivirals and vaccines.

Severe acute respiratory syndrome coronavirus 2 (SARS-CoV-2) is responsible for the ongoing coronavirus disease 2019 (COVID-19) pandemic that has caused more than 30 million infections, resulting in more than 900,000 deaths worldwide since December 2019 (https://covid19.who.int). Currently, there is an urgent need to better understand the molecular mechanisms governing SARS-CoV-2 pathogenesis, as this will have implications for the design of better treatments and vaccines.

Human coronaviruses are typically associated with mild upper respiratory illness. However, SARS-CoV-2 belongs to the Betacoronavirus genus, which also includes SARS-CoV (79% genetic similarity), and Middle East respiratory syndrome coronavirus (MERS-CoV) (about 50% similarity), and has the potential to infect both the upper and lower respiratory tracts leading to a severe and fatal respiratory syndrome in humans (1–4). Notably, severe infections with highly pathogenic coronaviruses are characterized by a dysregulated immune response leading to high levels of proinflammatory cytokines and extensive tissue damage (5–8).

Interferons (IFNs) are secreted cytokines with strong antiviral activities that constitute an important component of the first line of defense against invading pathogens. They are classified into three groups, type I, type II, and type III IFNs, based on the structure of their receptors on the cell surface (9, 10). Type I IFNs, or IFN-I, can be produced by virtually any nucleated cell type, and signal through the ubiquitously expressed type I IFN receptor (IFNAR). Type II IFN, or IFN-II, is mostly produced by specialized immune cells and signals through the IFN-γ receptor (IFNGR) to synergize innate and adaptive responses. Type III IFNs, or IFN-III, bind to the IFN-λ receptor (IFNLR), which is predominantly expressed in epithelial cells that are present at barrier surfaces such as the respiratory and gastrointestinal tracts. Type I and type III IFNs are induced upon sensing of different pathogen-associated molecular patterns (PAMPs) by different families of pattern recognition receptors (PRRs) and trigger a very similar antiviral response. Upon receptor binding, they both activate the JAK-STAT signaling cascade leading to the phosphorylation and activation of STAT1 and STAT2, which associate with IRF9 to form the IFN-stimulated gene factor 3 (ISGF3) transcriptional complex. ISGF3 is then imported into the nucleus by the karyopherin alpha 1 (KPNA1)-karyopherin beta 1 (KPNB1) heterodimer (11). Specifically, the karyopherins bind to nuclear localization signals exposed in the ISGF3 complex and mediate its cytoplasmic-nuclear translocation through interactions with proteins of the nuclear pore complex (NPC) termed nucleoporins or Nups. Once in the nucleus, ISGF3 binds to specific IFN-stimulated response elements (ISREs) in the DNA to trigger the transcription of IFN-stimulated genes (ISGs) and the establishment of an antiviral state. In contrast, JAK signaling downstream of IFNGR leads to phosphorylation and homodimerization of STAT1. STAT1 homodimer complexes then translocate into the nucleus as described above and regulate expression of a subset of ISGs by binding to gamma-activated site (GAS) promoter elements.

The role of IFNs and the kinetics of IFN secretion in the context of infection with highly pathogenic coronaviruses remain to be fully elucidated. As described for SARS patients, low levels of IFNs, accompanied by high levels of chemokines, have been detected in the blood and lung of patients with severe COVID-19 (5). However, an elevated IFN signature has been observed in the bronchoalveolar lavage (BAL) of some severe patients (8). Furthermore, in a mouse model of SARS-CoV-2 infection, IFN-I signaling appeared to be required for ISG induction and for the recruitment of proinflammatory cells into the lung, but was not effective at controlling virus replication (12), indicating that SARS-CoV-2 might be resistant to IFN signaling, as previously shown for SARS-CoV (6). This is likely due to the sophisticated mechanisms coronaviruses have evolved to evade and suppress the IFN response (13–16). For instance, coronaviruses are known to block IFN production by shielding their dsRNA intermediates of replication within double membrane vesicles (17) or by modifying viral mRNA to prevent recognition by specific PRRs (17–19). In addition, multiple betacoronavirus proteins have been shown to antagonize IFN signaling (20–24) or ISG effector functions (25, 26). For instance, the nonstructural protein Nsp1 of SARS-CoV was shown to decrease phosphorylation levels of STAT1 (22); the accessory proteins Orf3b and Orf6 appeared to inhibit IFN production and signaling by blocking nuclear translocation of transcription factors (20, 21); and the SARS-CoV-2 encoded papain-like protease antagonizes the action of the IFN-induced gene ISG15 (27).

In this study we investigated the ability of SARS-CoV-2 to antagonize IFN signaling. In agreement with recent studies, we found that SARS-CoV-2 is sensitive to IFN pretreatment (28). In addition, we show that viral infection is able to inhibit STAT nuclear import to impair transcriptional induction of ISGs. As shown with SARS-CoV (21), and more recently with SARS-CoV-2 (23, 24), expression of the viral accessory protein Orf6 is sufficient to inhibit STAT nuclear translocation. Mechanistically, we demonstrate that the accessory protein Orf6 localizes at the NPC where it binds directly to the Nup98-Rae1 complex to mediate this inhibition. Indeed, a mutant Orf6 protein defective in Nup98 binding completely loses the ability to block STAT nuclear translocation. Furthermore, we show that the interaction between Orf6 and Nup98 results in inefficient docking of cargo-receptor complexes at the NPC. Subversion of the IFN signaling pathway likely promotes unchecked SARS-CoV-2 replication in vivo and contributes to immunopathology.

## Results

### SARS-CoV-2 Is Sensitive to IFN Pretreatment.

**Fig. 1.**
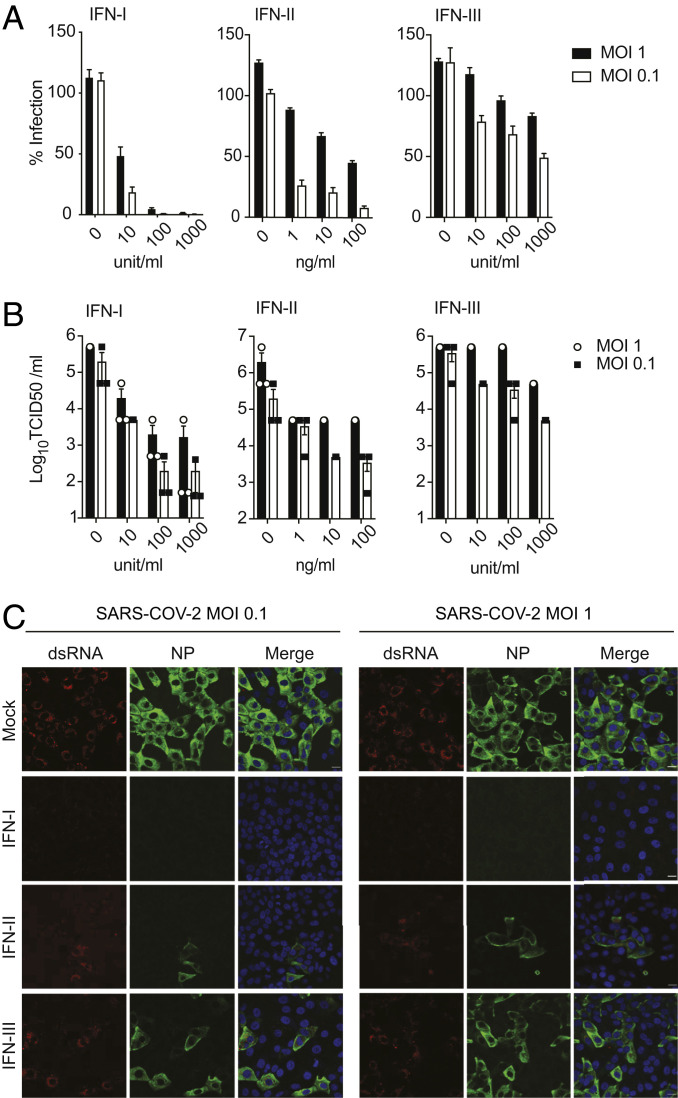
SARS-CoV-2 is sensitive to IFN pretreatment. (*A*) Vero E6 cells were treated for 16 h with the indicated concentrations of IFN-I, IFN-II, or IFN-III followed by infection with SARS-CoV-2 at the indicated MOI. After 24 of infection, cells were fixed and immunostained using antibodies against NP with a DAPI counterstain. The percentage of infected cells was calculated as the ratio of NP-positive versus total cells using a Celigo imaging cytometer. Data are represented as average ± SD (*n* = 3). (*B*) Supernatants from *A* were collected and used to assess changes in viral titers by TCID50 assay. Data are represented as average ± SEM (*n* = 3). (*C*) Confocal microscopy images of Vero E6 cells pretreated for 16 h with IFN-I (1,000 U/mL), IFN-II (100 ng/mL), or IFN-III (1,000 U/mL), and subsequently infected with SARS-CoV-2 at the indicated MOI. At 24 h postinfection, cells were stained with antibodies against NP or against the dsRNA intermediate of replication. (Scale bars, 20 µm.)

IFNs are a family of glycoproteins with essential roles in restricting viral replication and in modulating the antiviral immune response. To evaluate the susceptibility of SARS-CoV-2 to IFN pretreatment, Vero E6 cells were treated with different concentrations of type I, type II, or type III IFN for 16 h prior to infection. At 24 h postinfection, supernatants were collected to measure viral production via median tissue culture infectious dose (TCID50) assay, and cells were fixed to assess SARS-CoV-2 replication by immunostaining for the viral nucleoprotein (NP) and double-stranded RNA (dsRNA). Vero E6 cells were used in these studies, as they support robust replication of SARS-CoV-2 and, even though they cannot secrete type I IFN, they are able to respond normally to recombinant IFN when added to their culture media. As previously shown (28), we observed that IFN-I pretreatment drastically reduces the percentage of infected cells as well as viral titers in the supernatants, as compared to untreated cells (Fig. 1 *A–C*). In addition, we also observed that pretreatment with IFN-II inhibited viral NP expression in a dose-dependent manner, indicating that IFN-γ has significant anti-SARS-CoV-2 activity and can inhibit viral protein expression and replication in vitro (Fig. 1 *A–C*). However, pretreatment of Vero E6 cells with IFN-III resulted in only a minor reduction in the number of infected cells and infectious virus released in the culture media (Fig. 1 *A–C*). As IFN-III pretreatment effectively controlled SARS-CoV-2 infection in vitro and in vivo (29, 30), these results suggest that Vero E6 cells express only low levels of the IFN-III receptor.

### SARS-CoV-2 Antagonizes IFN Signaling.

**Fig. 2.**
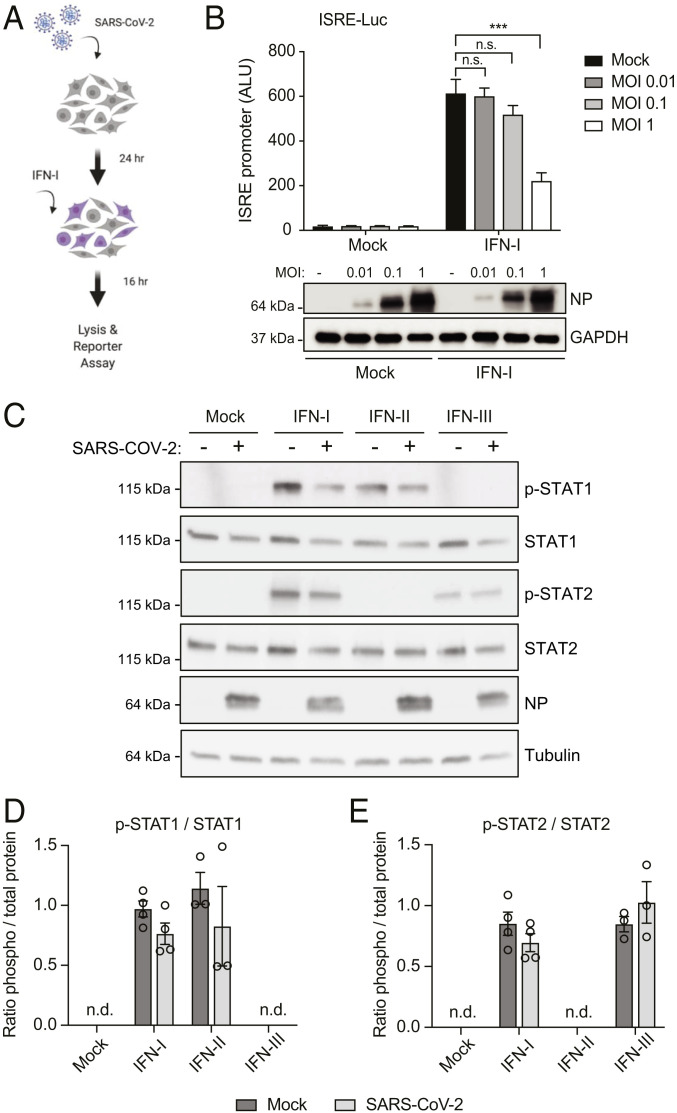
SARS-CoV-2 infection blocks IFN signaling downstream of STAT phosphorylation. (*A*) Schematic of the experimental setup. (*B*) hACE2-293T-ISRE-GF cells were seeded in 96-well format and infected with SARS-CoV-2 at the indicated MOI. After 24 h of infection, cells were stimulated with IFN-I (100 U/mL) for 16 h and then lysed to measure luciferase activity. Data are represented as average ± SD (*n* = 3). Significance was determined by unpaired two-tailed *t* test: *P* > 0.05 = n.s.; *P* < 0.001 = ***. ALU, absolute light unit. Cell lysates from the reporter assay were analyzed by Western blot to show NP expression. GAPDH was used as loading control. (*C*) Vero E6 cells were infected with SARS-CoV-2 at MOI 2 for 24 h and then treated with either IFN-I (1,000 U/mL), IFN-II (100 ng/mL), or IFN-III (1,000 U/mL) for 45 min. Expression of the indicated protein was determined by Western blot using tubulin as a loading control. (*D* and *E*) Western blot signals for phosphorylated STAT1 (*D*) and STAT2 (*E*) were quantified and compared to the corresponding total STAT1/STAT2 levels. Graphs show the mean p-STAT/STAT ratio and SEM from at least three independent experiments. n.d., not detected.

Many pathogenic viruses, including SARS-CoV and MERS-CoV, encode inhibitors of the type I IFN system (13, 31, 32). In order to investigate if SARS-CoV-2 has evolved mechanisms to counteract IFN-I signaling, we monitored IFN-induced, ISRE-dependent gene expression in mock versus virus-infected cells. For this experiment, HEK293T-ISRE reporter cells stably expressing human Ace2 (*SI Appendix*, Fig. S1) were infected at three different multiplicities of infection (MOIs). Twenty-four hours postinfection, cells were treated with 100 U/mL of IFN-I for 16 h to trigger activation of the promoter and then analyzed for firefly luciferase expression (Fig. 2*A*). As expected, treatment with IFN-I strongly activated the ISRE promoter in uninfected control cells. However, SARS-CoV-2-infected cells suppressed IFN-induced, ISRE-dependent gene expression in a MOI-dependent fashion (Fig. 2*B*). As shown in *SI Appendix*, Fig. S1*C*, only 40 to 50% of the reporter cells are productively infected at a MOI of 1, and uninfected cells in the well would still be able to activate the ISRE promoter in response to IFN-I treatment. This may explain why the block of IFN signaling in this assay is not complete. Next, to investigate the mechanism by which SARS-CoV-2 antagonizes IFN signaling, we monitored STAT1 and STAT2 expression and phosphorylation levels by Western blot in mock versus infected Vero E6 cells, 45 min after IFN treatment. As shown in Fig. 2*C*, type I IFN treatment resulted in the phosphorylation of both STAT1 and STAT2, while type II IFN only triggered STAT1 phosphorylation as expected. Intriguingly, we found that viral infection did not impact STAT1 and STAT2 expression and only slightly decreased IFN-I-mediated STAT phosphorylation (Fig. 2 *D* and *E*). Like IFN-I, IFN-III is known to activate Jak1 and Tyk2, leading to the phosphorylation and nuclear translocation of STAT1 and STAT2. However, in our assay we could not detect STAT1 phosphorylation, and we could only observe weak STAT2 phosphorylation upon IFN-λ1 treatment. This is consistent with the modest antiviral activity of IFN-III that we observed in Vero E6 (Fig. 1 *A*–*C*). As with IFN-I treatment, SARS-CoV-2 infection did not impact IFN-III-mediated phosphorylation of STAT2. This suggests that SARS-CoV-2 predominantly suppresses IFN signaling downstream of STAT phosphorylation, as it was previously shown for SARS-CoV (21).

### SARS-CoV-2 Infection Inhibits Nuclear Translocation of STAT1 and STAT2.

**Fig. 3.**
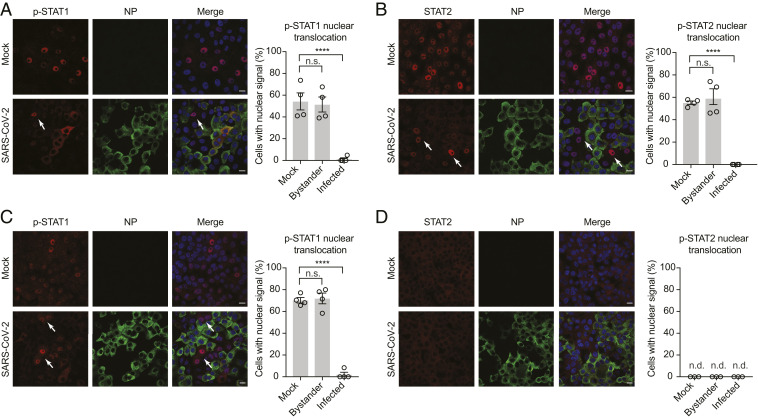
SARS-CoV-2 infection prevents nuclear translocation of STAT1 and STAT2. (*A* and *B*) Vero E6 cells were mock infected or infected with SARS-CoV-2 at an MOI of 2. At 24 h postinfection, cells were either mock treated or treated with IFN-I (1,000 U/mL) for 45 min. The subcellular localization of the indicated protein was analyzed by confocal microscopy. Nuclei were stained with DAPI. STAT nuclear translocation in infected and bystander cells was quantified from ≥200 cells per condition from two biological replicates and compared to translocation in mock-infected cells. Data are shown as average ± SEM. Significance was determined by unpaired two-tailed *t* test: *P* > 0.05 = n.s.; *P* < 0.0001 = ****. (*C* and *D*) Vero E6 cells were infected as described in *A* and then stimulated with IFN-II (100 ng/mL). The subcellular localization of the indicated protein was analyzed by confocal microscopy and quantified as described in *A*. Nuclei were stained with DAPI. White arrows show STAT translocation in bystander cells. (Scale bars, 20 µm.) n.d., not detected.

Next, we investigated the effect of SARS-CoV-2 infection on STAT1 and STAT2 subcellular localization and nuclear translocation in response to type I and type II IFN. Vero E6 cells were infected at a MOI of 2 for 24 h and then treated for 45 min with the two IFNs before fixing them for indirect immunofluorescence assay (IFA). Active STAT1 and STAT2 must be phosphorylated and translocate into the nucleus in order to function as signal transducers of transcription and up-regulate hundreds of ISGs. As seen in Fig. 3 *A* and *C*, pSTAT1 is efficiently translocated into the nucleus in mock-infected cells upon IFN-I and IFN-II treatment (55% and 70%, respectively). However, SARS-CoV-2 infection clearly prevented STAT1 nuclear translocation. Interestingly, only a fraction of the IFN-treated infected cells (∼40% of cells treated with IFN-I, and 15% of cells treated with IFN-II) showed accumulation of phosphorylated STAT1 (p-STAT1) in the cytoplasm. This is consistent with the slightly decreased levels of p-STAT1 detected in Fig. 2*C* and suggests that SARS-CoV-2 may be able to antagonize IFN signaling at multiple levels. Likewise, IFN-I-mediated STAT2 nuclear translocation was also dramatically blocked in productively infected Vero E6 cells (Fig. 3*B*). Next, we assessed whether the same nucleocytoplasmic trafficking defect was also observed in hAce2-HEK293T and Calu3, a human bronchial epithelial cell line that is known to support SARS-CoV-2 replication (33). In agreement with our findings in Vero E6 cells, SARS-CoV-2 infection clearly impaired STAT2 nuclear translocation in IFN-I-treated hAce2-HEK293T and Calu-3 cells (*SI Appendix*, Fig. S2). All together these observations confirm that SARS-CoV-2 infection suppresses IFN signaling downstream of STAT phosphorylation and disrupts nucleocytoplasmic trafficking of host immune signaling factors to promote viral replication.

### SARS-CoV-2 Infection Inhibits IFN-Dependent ISG Induction.

**Fig. 4.**
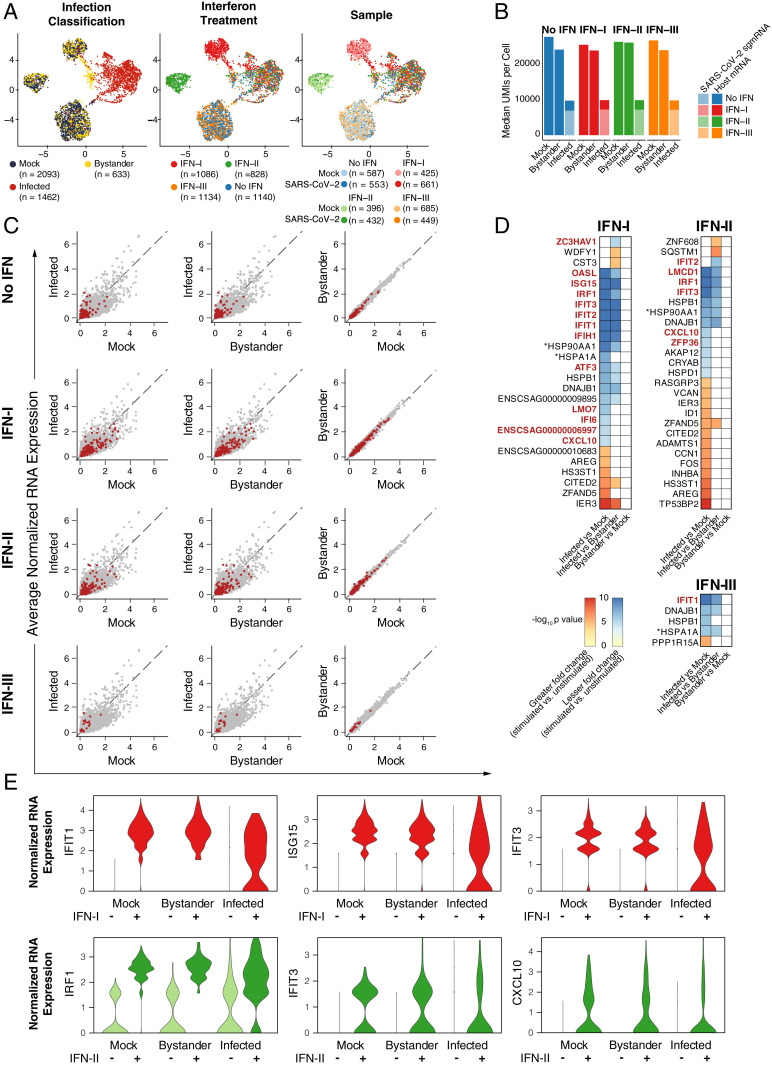
scRNA-Seq of SARS-CoV-2 infected, uninfected, and bystander cells. (*A*) Uniform manifold approximation and projection (UMAP) plots of Vero E6 cells treated with IFN-I, IFN-II, and IFN-III in the presence or absence of SARS-CoV-2. Points are colored by infection status as classified by SARS-CoV-2 sgmRNA expression (*Left*), IFN treatment (*Center*), or sample of origin (*Right*). (*B*) Median total number of transcript UMIs per cell derived from SARS-CoV-2 or from host genes by infection status and IFN condition. (*C*) Scatterplots of average normalized gene expression for all expressed genes in indicated conditions. Each row compares infected, mock, or bystander cells within an IFN condition. Red points indicate ISGs from all IFN treatments (*Top*) or from corresponding IFN treatment (IFN-I, IFN-II, or IFN-III, respectively, from the second row). Normalized expression values were calculated from downsampled transcript count data (to mitigate the effects of different host gene detection rates due to infection status, see *Materials and Methods*). (*D*) Genes with significant differential induction (IFN-I, IFN-II, or IFN-III stimulated versus unstimulated) between infection status groups (mock, bystander, and infected). *Gene ID ENSCSAG00000010250 and ENSCSAG00000019310 are labeled HSP90AA1 and HSPA1A, respectively, based on National Center for Biotechnology Information (NCBI)/Ensembl annotations. (*E*) Normalized expression (downsampled transcript counts) for several ISGs with significant differences in induction between infection status groups.

To test whether SARS-CoV-2 infection interferes with the induction of endogenous ISGs, we performed single-cell RNA sequencing (scRNA-Seq) on Vero E6 cultures that were infected or mock infected and later stimulated with IFN-I, IFN-II, or IFN-III. Within infected cultures, cells were classified as infected or bystander based on expression of 10 viral subgenomic mRNA (sgmRNA) species (*SI Appendix*, Fig. S3). This approach enabled the direct comparison of infected cells and bystander cells within the same culture, as well as uninfected cells from mock cultures. We detected significantly lower total and host gene transcript counts (as quantified by unique molecular identifiers [UMIs]) in infected cells than bystander or mock cells (Fig. 4*B*). Remarkably, and in agreement with previous observations (34), within the already reduced total transcript counts of infected cells, 73% (median) of detected transcripts were from SARS-CoV-2. These data suggest that, in Vero E6 cells infected with SARS-CoV-2, the cellular transcriptome is primarily derived from viral transcription. We next conducted differential expression across infection conditions. To mitigate the effects of different host gene detection rates due to infection status, transcript counts were randomly downsampled to equivalent levels for comparison. Within the remaining host gene-derived transcriptome, we observed significantly reduced expression of numerous genes in infected cells compared to bystander and mock cells (Fig. 4*C* and Datasets S2–S5 and S9 and *SI Appendix*, Fig. S8). These data are consistent with widespread transcriptional dysregulation and host gene suppression by SARS-CoV-2 infection. We further explored the effects of SARS-CoV-2 infection on the expression of ISGs induced by IFN-I, IFN-II, or IFN-III. While IFN-I and IFN-II stimulation induced robust expression of ISGs in mock and bystander cells (*SI Appendix*, Fig. S4 and Dataset S1), in infected cells most ISGs were among those genes expressed at lower levels (relative expression within host genes as described above, Fig. 4*C* and Dataset S1). Furthermore, in differential expression statistical testing of the response to IFN (i.e., IFN stimulated versus unstimulated between different infection conditions), the induction of several IFN-I and IFN-II ISGs was significantly blunted in infected cells (Fig. 4 *D* and *E* and Dataset S6–S8). Our scRNA-Seq analysis suggests that cells infected with SARS-CoV-2 do not mount a transcriptional response comparable to bystander or mock cells when stimulated with IFN-I or IFN-II. In comparison to IFN-I and IFN-II, IFN-III induces minimal expression of ISGs in Vero E6 cells, even in the absence of infection (Fig. 4*A*, *SI Appendix*, Fig. S4, and Dataset S1). This may explain the modest antiviral activity of IFN-III treatment in Vero E6 cells.

### Ectopic Expression of SARS-CoV-2 Orf6 Recapitulates IFN Signaling Blockage.

**Fig. 5.**
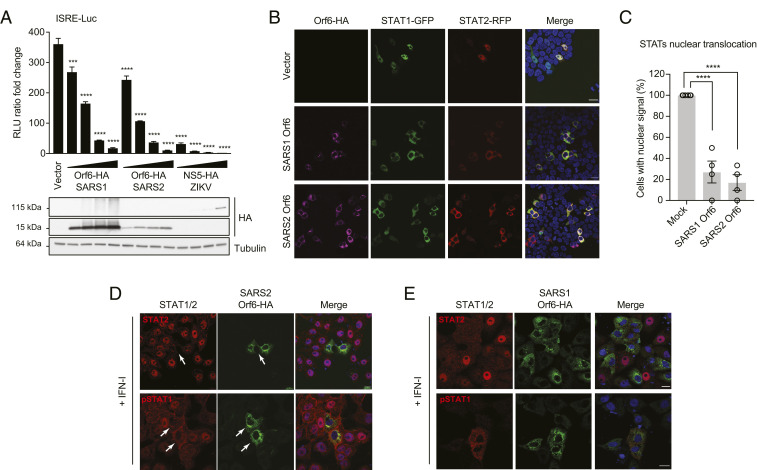
SARS-CoV-2 Orf6 inhibits IFN signaling by preventing nuclear translocation of STAT1 and STAT2. (*A*) HEK293T cells were transiently transfected with plasmids expressing the indicated viral proteins (0.5 ng, 2 ng, 5 ng, or 10 ng), a plasmid encoding an ISRE-firefly luciferase reporter, and plasmid expressing Renilla luciferase from the TK promoter. At 24 h posttransfection, cells were treated with IFN-I (1,000 U/mL) for 16 h prior to measuring luciferase activities. Data are representative of three independent experiments and shown as average ± SD (*n* = 3). Significance was determined by unpaired two-tailed *t* test: *P* < 0.001 = ***; *P* < 0.0001 = ****. Cell lysates from the reporter assay were analyzed by Western blot to show relative expression of each transfected viral protein. Tubulin was used as loading control. (*B*) Confocal microscopy images of HEK293T cells ectopically expressing SARS-CoV (SARS1) or SARS-CoV-2 (SARS2) Orf6 along with STAT1-GFP and STAT2-RFP. At 24 h posttransfection, cells were mock stimulated or stimulated for 45 min with IFN-I (1,000 U/mL) to monitor STAT1 and STAT2 subcellular localization. (*C*) Quantification of nuclear translocation of ectopically expressed STAT1 and STAT2 in control and Orf6-positive cells from four fields of view collected from two independent experiments. Data are shown as average ± SEM. Significance was determined by unpaired two-tailed *t* test: *P* < 0.0001 = ****. (*D* and *E*) Confocal microscopy images of IFN-I-treated Vero E6 cells ectopically expressing SARS-CoV-2 (*D*) or SARS-CoV (*E*) Orf6-HA. The subcellular localization of endogenous STAT2 and phosphorylated STAT1 (pSTAT1) is shown. (Scale bars, 20 µm.)

Orf6 of SARS-CoV, and more recently Orf6 from SARS-CoV-2, has been shown to prevent STAT translocation (21, 23, 24). Consistent with these studies, we show that SARS-CoV-2 Orf6 overexpression was able to suppress ISRE-dependent gene expression in response to recombinant IFN-I. HEK293T cells were transiently transfected with an ISRE-firefly luciferase reporter plasmid together with increasing amounts of a SARS-CoV-2 Orf6 expression construct and a constitutively expressed Renilla luciferase plasmid for normalization. SARS-CoV Orf6 and ZIKV NS5 expression vectors were used as controls for this experiment. Strikingly, expression of each of the three viral proteins suppressed IFN-dependent ISRE induction in a dose-dependent manner, indicating that SARS-CoV-2 Orf6 can block IFN signaling as effectively as SARS-CoV Orf6 in this assay (Fig. 5*A*). To determine whether Orf6 overexpression could recapitulate the block of STAT nuclear import observed during infection, HEK293T cells were transfected with STAT1-GFP and STAT2-RFP fusion constructs together with plasmids expressing SARS-CoV or SARS-CoV-2 Orf6. Twenty-four hours posttransfection, cells were treated for 45 min with IFN-I, fixed, and analyzed by confocal microscopy. As expected, IFN-I treatment triggered STAT1-GFP and STAT2-RFP nuclear translocation in empty vector-transfected cells. However, expression of the Orf6 protein from either SARS virus dramatically blocked their nuclear translocation (Fig. 5 *B* and *C*). Importantly, this phenotype was also confirmed when we looked at the localization of endogenous STAT2 and phosphorylated STAT1 in Orf6-expressing Vero E6 cells (Fig. 5 *D* and *E*). Following IFN stimulation, STAT1:STAT1 or STAT1:STAT2 dimers translocate into the nucleus by binding to the import receptor karyopherin alpha 1, KPNA1 (11). The STAT:KPNA1 complex then interacts with KPNB1, which mediates docking of the import complex to the NPC (35–38). A previous study has shown that SARS-CoV Orf6 interferes with IFNAR signaling by tethering KPNA2 and KPNB1 to the endoplasmic reticulum (ER)/Golgi membrane to block STAT1 nuclear import (20, 21). Therefore, we investigated whether SARS-CoV-2 Orf6 might also interact with KPNA2 or other nuclear import factors to block IFN signaling. For this purpose, we performed coimmunoprecipitation assays with lysates from HEK293T cells overexpressing Orf6, KPNA1, and KPNA2. We found that Orf6 interacts with both KPNA1 and KPNA2 (*SI Appendix*, Fig. S5 *A* and *B*). In addition, confocal immunofluorescence analysis revealed a change in localization of KPNA1 and KPNA2 from the nucleus to the cytoplasm in Orf6-expressing cells (*SI Appendix*, Fig. S5 *C* and *D*). However, overexpression of KPNA1 or KPNA2 could not rescue the Orf6-dependent block of STAT1-GFP nuclear translocation (*SI Appendix*, Fig. S5 *E* and *F*), suggesting that karyopherin alphas may not be the specific factors directly targeted by SARS-CoV-2 to block IFN signaling.

### SARS-CoV-2 Orf6 Directly Interacts with the Nup98-Rae1 Complex.

**Fig. 6.**
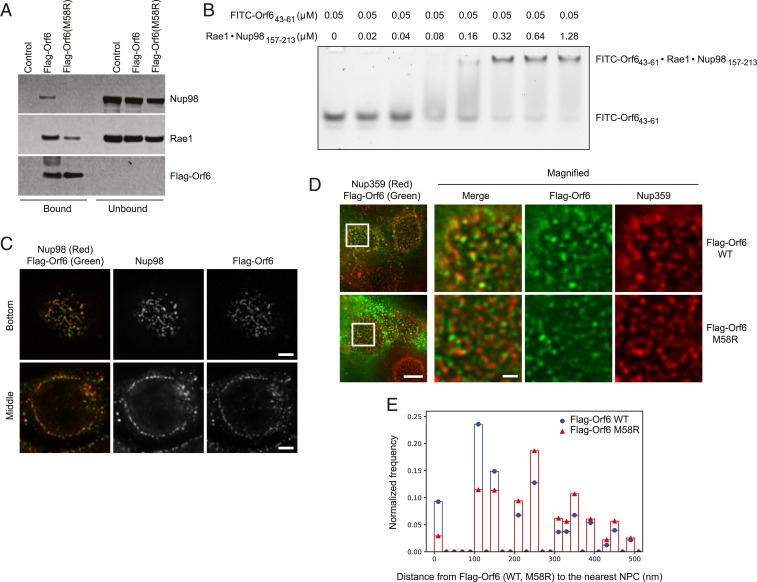
SARS-CoV-2 Orf6 directly interacts with Nup98-Rae1. (*A*) HEK293T cells were transfected for 24 h with Flag-Orf6, Flag-Orf6_M58R_, or vector control. Cell lysates were subjected to IP using anti-Flag antibody and followed by immunoblot (IB) with antibodies against the depicted proteins. (*B*) EMSA was carried out with 0.05 µM FITC-labeled Orf6 (43–61) and increasing concentrations of purified Rae1•Nup98(157-213) complex as indicated. (*C*) HEK293T cells were transfected with Flag-Orf6 and costained with anti-Flag and anti-Nup98 antibodies. Representative images from a z-stack obtained using STED microscopy imaging are shown. (*D*) HEK293T cells were transfected with Flag-Orf6 or Flag-Orf6_M58R_ and costained with anti-Flag and anti-Nup358 antibodies. The optical sections containing a flat surface of the nuclear envelope (the bottom of the nucleus) are presented. The regions indicated by the white rectangles are magnified and shown at *Right*. (Scale bars in the full field and the magnified images, 5 μm and 1 μm, respectively.) (*E*) The distance from each Flag-Orf6 or Flag-Orf6_M58R_ spot to the nearest NPC spot was calculated, and the distribution of the measured distances is presented as a histogram with a bin width of 20 nm. The *y* axis represents the frequency relative to the total number of the distance measurements. Flag-Orf6 *n* = 22 nuclei; Flag-Orf6_M58R_ *n* = 21 nuclei.

A global analysis of SARS-CoV-2 host interacting proteins recently identified the Nup98-Rae1 complex as a high-confidence SARS-CoV-2 Orf6 binding partner (39). Therefore, we sought to investigate whether this interaction could contribute to the IFN signaling blockage. To validate this interaction, we ectopically expressed Flag-Orf6 in HEK293T cells and performed immunoprecipitations (IPs) with an anti-Flag antibody. As shown in *SI Appendix*, Fig. S6*A*, both Nup98 and Rae1 coprecipitated with Orf6; however, no interactions were observed in extracts of cells transfected with an empty vector control. In addition, RNase treatment did not impair the ability of Orf6 to associate with the Nup98-Rae1 complex, demonstrating that RNA is not required for this interaction. We previously found that the finger region of the matrix protein of vesicular stomatitis virus (VSV M) (residues 49 to 61) is critical for binding to the Nup98-Rae1 heterodimer (40, 41). In addition, several studies have shown that the highly conserved methionine at residue 51 (Met51) is important for VSV M function (42, 43). Interestingly, a similar Nup98-Rae1 interaction motif is also present in the C-terminal sequence of both SARS-CoV and SARS-CoV-2 Orf6 (39). Prompted by this observation, we investigated whether a methionine-to-arginine substitution at residue 58 (M58R) was able to affect Orf6 binding to the Nup98-Rae1 complex. Strikingly, the M58R mutation dramatically decreased the ability of SARS-CoV-2 Orf6 to interact with overexpressed (*SI Appendix*, Fig. S6*B*) and endogenous Nup98 (Fig. 6*A*) without impairing KPNA1 or KPNA2 binding (*SI Appendix*, Fig. S6 *C* and *D*). Next, we tested whether SARS-CoV-2 Orf6 is able to bind purified recombinant Nup98-Rae1 complex using an electrophoretic mobility shift assay (EMSA). Indeed, the complex of full-length Rae1 and the Rae1 binding motif in Nup98 (residues 157 to 213) reduced the mobility of fluorescein isothiocyanate (FITC)-labeled Orf6 (residues 43 to 61) in a concentration-dependent manner (Fig. 6*B*). This result demonstrates that the C-terminal region of Orf6 directly binds the Nup98-Rae1 complex with an estimated dissociation constant in the submicromolar range. The assays described above clearly demonstrate that SARS-CoV2 Orf6 and the Nup98-Rae1 complex interact in vitro. Nup98 is a peripheral nucleoporin that localizes at both sides of the NPC in a distinct punctate pattern along the surface of the nucleus. In addition, Nup98 can also localize within the nucleus, or associate with annulate lamellae in the cytoplasm (44–46). To further evaluate the in situ association of Nup98 with Orf6, we expressed Flag-Orf6 in HEK293T cells and compared their localization pattern by immunofluorescence microscopy. Consistent with their interactions, stimulated emission depletion (STED) superresolution microscopy showed extensive colocalization of the two proteins, both along the nuclear periphery and at cytoplasmic foci (Fig. 6*C* and Movie S1). Moreover, Flag-Orf6 showed a similar colocalization with another NPC protein, Nup358 (Fig. 6*D* and Movie S2). Interestingly, when we compared the relative position of Flag-Orf6 and Flag-Orf6_M58R_ to NPCs detected using Nup358 antibodies, we found that the majority of Orf6 signal was <160 nm from NPCs consistent with its association with the NPCs, while the Flag-Orf6_M58R_ foci were broadly distributed across the nuclear envelope (Fig. 6*E*). Together, these results lead us to conclude that Orf6 is likely associated with Nup98 both at NPCs present at the nuclear envelope and at annulate lamellae in the cytoplasm. These data are consistent with Orf6 altering nuclear transport functions of Nup98 at NPCs.

### Nup98 Binding to SARS-CoV-2 Orf6 Induces Block of STAT1 Nuclear Translocation.

**Fig. 7.**
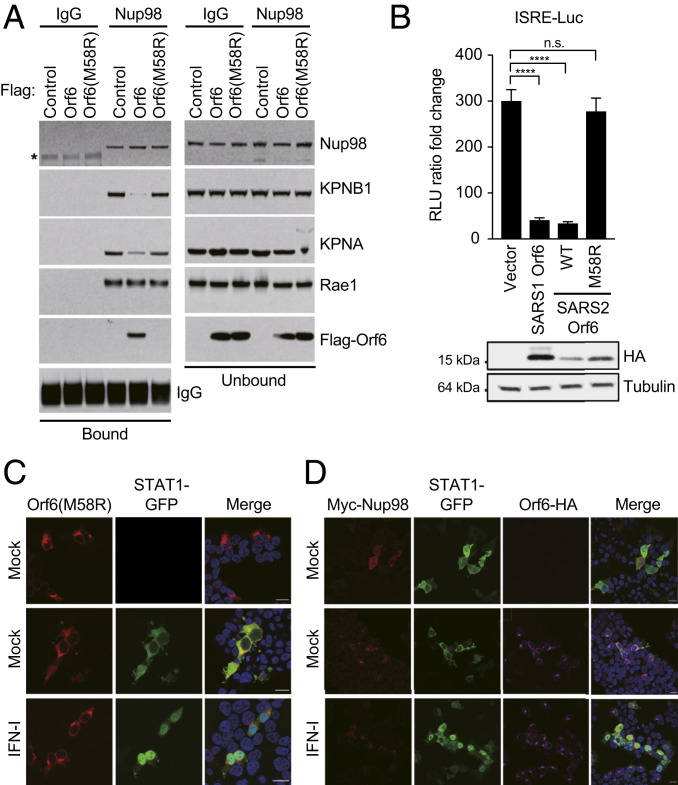
The Orf6-Nup98 interaction is critical to block STAT nuclear translocation and IFN signaling. (*A*) HEK293T cells were transfected for 24 h with Flag-Orf6, Flag-Orf6_M58R_, or vector control. Cell lysates were subjected to IP using anti-Nup98 antibody, or an isotype control (IgG), and followed by immunoblot with the antibodies indicated in the figure. (*B*) HEK293T cells were transiently transfected with plasmids expressing the indicated viral proteins, a plasmid encoding an ISRE-firefly luciferase reporter, and plasmid expressing Renilla luciferase from the TK promoter. At 24 h posttransfection, cells were treated with IFN-I (1,000 U/mL) for 16 h prior to measuring luciferase activities. Data are representative of three independent experiments and shown as average ± SD (*n* = 3). Significance was determined by unpaired two-tailed *t* test. *P* > 0.05 = n.s. ; *P* < 0.0001 = ****. Cell lysates from the reporter assay were analyzed by Western blot to show relative expression of each transfected viral protein. Tubulin was used as loading control. (*C*) Confocal microscopy images of HEK293T cells ectopically expressing SARS-CoV-2 Orf6_M58R_-HA along with STAT1-GFP. Cells were either mock stimulated or stimulated for 45 min with IFN-I (1,000 U/mL) prior to fixation in order to assess nuclear translocation of STAT1-GFP. (*D*) Confocal microscopy images of HEK293T cells ectopically expressing SARS-CoV-2 Orf6-HA along with Myc-Nup98 and STAT1-GFP. Cells were either mock stimulated or stimulated for 45 min with IFN-I (1,000 U/mL) prior to fixation in order to assess nuclear translocation of STAT1-GFP. (Scale bars, 20 µm.)

To determine if the interaction of Orf6 with Nup98 might account for the impaired nuclear translocation of STAT1 and STAT2, we investigated if Orf6 is able to disrupt the Nup98-KPNB1 interaction. HEK293T cells were transfected with empty vector control plasmid or with plasmids expressing either Flag-Orf6 or Flag-Orf6_M58R_. After 24 h, cell lysates were subjected to immunoprecipitations with an anti-Nup98 antibody (45). As shown in Fig. 7*A*, the KPNB1-KPNA complex coprecipitated with Nup98 and Rae1 in extracts of cells transfected with a control plasmid. Furthermore, in agreement with the Flag-Orf6 immunoprecitation shown in Fig. 6*A*, ectopically expressed Flag-Orf6, but not Flag-Orf6_M58R_, formed a complex with Nup98 and Rae1. Notably, Flag-Orf6 expression specifically disrupted the interaction of Nup98 with the KPNB1-KPNA complex without affecting Rae1 binding. These results suggest that Orf6 binding to Nup98 impairs docking of KPNB1-KPNA complexes at the NPC to target the nuclear import pathway. Next, we tested whether Nup98 binding is required to inhibit IFN signaling. HEK293T cells were transfected with an ISG54 reporter vector along with a plasmid constitutively expressing Renilla luciferase and empty vector or Orf6 expressing plasmids as indicated in Fig. 7*B*. After 24 h, cells were treated with IFN-I for 16 h prior to measuring luciferase activities. While wild-type SARS-CoV and SARS-CoV-2 Orf6 strongly inhibited the ISRE promoter as expected, the M58R mutation completely abolished the Orf6 IFN antagonistic function (Fig. 7*B*). In agreement with these data, ectopic expression of the Orf6_M58R_ mutant was not able to block STAT1-GFP nuclear translocation upon IFN treatment (Fig. 7*C*). Next, because the Orf6_M58R_ mutant was still able to interact with KPNA1 and KPNA2 in our coimmunoprecipitation assays, we asked whether ectopic expression of Orf6_M58R_ could alter their intracellular localization. As shown in *SI Appendix*, Fig. S7, both KPNA1 and KPNA2 localized to the nucleus in Orf6_M58R_-expressing cells, indicating that the SARS-CoV-2-dependent cytoplasmic accumulation of the KPNAs is likely a result of the impaired nucleocytoplasmic trafficking due to the Orf6-Nup98 interaction. Finally, to determine whether Nup98 is the critical factor hijacked by SARS-CoV-2 to inhibit IFN signaling, we tested whether Nup98 overexpression is able to revert the Orf6-mediated inhibition of STAT1 nuclear translocation. As shown in Fig. 7*D*, we observed a complete rescue of STAT1 translocation in Nup98 overexpressing cells, strongly suggesting that Orf6 specifically targets Nup98 to block STAT nuclear import.

## Discussion

Highly pathogenic coronaviruses have evolved multiple strategies to suppress the IFN response and successfully replicate in host cells. In this study, we show at a single-cell resolution, that SARS-CoV-2 infection strongly inhibits type I and type II IFN signaling by blocking STAT1 and STAT2 nuclear translocation (Fig. 3) to dampen ISG induction (Fig. 4). The nuclear localization of STAT1 and STAT2 is triggered by their tyrosine phosphorylation-mediated homo- (IFN-II mediated) and heterodimerization (IFN-I- and IFN-III mediated) (47). STAT1 homodimers and STAT1-STAT2 heterodimers are then transported into the nucleus by the KPNA1-KPNB1 complex, which is required for docking the import complex to the NPC (48). It was previously proposed that SARS-CoV blocks IFN signaling through both an nsp1-mediated inhibition of STAT1 phosphorylation (22) and an Orf6-dependent block of STAT1 nuclear translocation (20, 21). Our data demonstrate that SARS-CoV-2 infection only marginally impairs STAT1 and STAT2 phosphorylation (Fig. 2 *C*–*E*), suggesting that the virus is primarily targeting host factors that are more downstream in the pathway to block IFN signaling. Whether SARS-CoV-2 nsp1 is responsible for the observed decrease in STAT1 phosphorylation remains to be determined. However, in agreement with recent reports (23, 24), we found that ectopic expression of the SARS-COV-2 accessory protein Orf6 was able to alter STAT nucleocytoplasmic trafficking and to suppress ISRE-dependent gene expression in response to recombinant IFN-I (Fig. 5). Hence, despite having only 69% amino acid identity, SARS-CoV and SARS-CoV-2 Orf6 can both effectively antagonize IFN signaling. Many viruses have been shown to target host transport pathways in order to subvert innate antiviral responses and promote viral replication (14, 49, 50). Yet, the mechanisms exploited by the different viruses appear to be diverse. While poliovirus (PV) and human rhinovirus (HRV) trigger proteolytic degradation of several nucleoporins (Nups), including Nup98, infection with encephalomyocarditis virus (EMCV) and mengovirus result in changes in their phosphorylation status (51–55). The Ebola virus (EBOV) VP24 protein binds KPNA1 to disrupt the formation of the IFN-mediated pSTAT1-KPNA1 complex and prevent STAT1 nuclear translocation (56). Furthermore, VSV M inhibits cellular mRNA export by forming a complex with Rae1 and Nup98 (41, 57). Lastly, as mentioned before, SARS-CoV Orf6 has been proposed to bind KPNA2 and sequester KPNB1 to the ER membrane in order to block STAT1 transport into the nucleus (21). Here we show a mechanism of viral antagonism in which a virus hijacks the Nup98-Rae1 complex to prevent STAT nuclear import. Like VSV M, SARS-CoV-2 Orf6 also forms a complex with Nup98 and Rae1 (Fig. 6). Here we show that this interaction appears to disrupt docking of the cargo-KPNA1-KPNB1 complexes at the NPC (Fig.7*A*). This is consistent with its ability to target the nuclear import pathway and to retain phosphorylated STAT1 and STAT2 into the cytoplasm. Based on the structure of VSV M with the Nup98-Rae1 complex, VSV M protein binds to Nup98-Rae1 through both its globular domain and through an extended finger region (40). Specifically, the finger of VSV M binds to the interface of Nup98 and Rae1, with its Met51 residue recognizing a hydrophobic pocket on the side of the Rae1 beta-propeller and the neighboring acidic residue-enriched segments interacting with both proteins. The Orf6 C-terminal region resembles the finger of VSV M, featuring the critical Met58 residue flanked by acidic residues. While Orf6 likely binds to Nup98-Rae1 in a similar manner to the finger of VSV M, Orf6 lacks the other interface mediated by the globular domain of VSV M. Since the karyopherin beta 1 site is close to the Rae1 binding site on Nup98, it is possible that ORF6 binding to Nup98 disrupts KPNB1 docking at Nup98, as our results indicate. In addition, the VSV M protein and the Nup98-Rae1 complex are found both inside the nucleus and at the nuclear pore complex (41, 58), while Orf6 is mostly found in the cytoplasm and at the NPC (Fig. 6 *C*–*E*). Therefore, while we cannot exclude that by interacting with the Nup98-Rae1 complex Orf6 may also have an effect on mRNA export, it is likely that the differences listed above would result to contribute to distinct effects of these proteins on nuclear transport. This is an interesting topic for future investigation.

Notably, a mutant Orf6 protein (methionine-to-arginine substitution at residue 58) that is deficient in Nup98 binding (Fig. 6*A*) did not suppress ISRE-dependent gene expression and STATs nuclear translocation (Fig. 7 *B* and *C*). Interestingly, this mutant Orf6 protein was still able to interact with KPNA1 and KPNA2 (*SI Appendix*, Fig. S7 *C* and *D*), suggesting that although the Orf6-KPNAs interaction may contribute to the retention of STAT1 and STAT2 into the cytoplasm, it is not sufficient to drive this phenotype. In addition, we show that Nup98 overexpression is able to fully reverse the Orf6-mediated block of STATs nuclear import (Fig. 7*D*). Taken together, these data demonstrate that Orf6 is a potent IFN antagonist and that Nup98 binding is critical for Orf6-mediated inhibition of IFN signaling. In addition to blocking STAT nuclear translocation, the observed Orf6-mediated perturbation of nuclear import might also affect other KPNB1 cargos and result in the alteration of a broader range of signaling networks. In this regard, it has been recently shown that SARS-CoV-2 Orf6 can also suppress IRF3 activation via its C-terminal tail (24). However, it still remains to be established whether Nup98 binding is required for this inhibition. In agreement with these findings, our scRNA-Seq analysis on Vero E6 cells strongly suggests that cells productively infected with SARS-CoV-2 do not mount a transcriptional response comparable to bystander or mock cells when stimulated with IFN-I or IFN-II (Fig. 4 and *SI Appendix*, Fig. S4). Importantly, our data also show that SARS-CoV-2 infection triggers a dramatic reduction of total RNA content in infected cells as compared to uninfected or bystander cells (Fig. 4*B*). This strongly suggests that viral infection results in extensive transcriptional dysregulation and host gene suppression that is likely due to the combined action of multiple viral factors, including Orf6. Nevertheless, the Orf6-mediated suppression of IFN signaling in infected tissues is likely to promote viral replication and to play an important role in the pathogenesis of COVID-19. Therefore, it will be important in the near future to address the contribution of Orf6 to the in vivo pathogenesis of SARS-CoV-2.

## Materials and Methods

Detailed methodology can be found in *SI Appendix*.

### Cells and Viruses.

Vero E6 (ATCC, CRL-1586), Calu3 (ATCC, HTB-55), and HEK293T (ATCC, CRL-3216; a kind gift from Viviana Simon, Icahn School of Medicine at Mount Sinai, New York, NY), were maintained in Dulbecco’s modified Eagle’s medium (Corning) supplemented with 10% fetal bovine serum (Peak Serum) and penicillin/streptomycin (Corning) at 37 °C and 5% CO_2_. hACE2-293T-ISRE-GF cells were generated for this study. Briefly, HEK293T cells were transduced with a lentiviral vector coexpressing GFP and firefly luciferase from ISRE transcriptional response elements paired with a minimal CMV promoter (pGreenFire1-ISRE; System Biosciences). Puromycin-resistant cells were single-cell cloned and screened for their responsiveness to recombinant IFN-I. A single-cell clone exhibiting good IFN sensitivity and high amplitude of IFN response was then transduced with a lentiviral vector expressing human ACE2 harboring a C-terminal HA-FLAG tag. Ace2-positive cells were sorted by fluorescence activated cell sorting after staining with Alexa Fluor 647-conjugated goat anti-hACE2 antibodies. All cell lines used in this study were regularly screened for *Mycoplasma* contamination using the Universal *Mycoplasma* Detection Kit (ATCC, 30-1012K). Cells were infected with SARS-CoV-2, isolate USA-WA1/2020 (BEI Resources NR-52281) under BSL3 containment in accordance with the biosafety protocols developed by the Icahn School of Medicine at Mount Sinai. Viral stocks were grown in Vero E6 cells as previously described (59) and validated by genome sequencing.

### IFN Pretreatment.

Vero E6 cells were seeded into 24-well glass bottom plates and treated with either universal IFN type I (1,000 U/mL), or IFN-gamma (100 ng/mL), or IFN-lambda 1 (1,000 U/mL) overnight. On the next day, cells were infected with SARS-CoV-2 at the indicated MOI in viral growth media. At 24 h after infection, supernatants were collected to measure viral titers by TCID50, and cells were fixed and immunostained with the indicated antibodies prior to image analysis. Infection rates were assessed using the protocol described elsewhere (59). A plate cytometer (Celigo) was used for accurate quantification of the percentage of infected cells in each condition. TCID50 assays were performed as described before (59). In brief, Vero E6 cells were infected with serial 10-fold dilutions of the supernatants harvested from cells pretreated with IFN at the concentrations indicated in the figures. On day 4 postinfection, cytopathic effect (CPE) was assessed by crystal violet staining, and TCID50 was calculated using the Reed and Muench method.

### Luciferase Assay.

For luciferase assays, HEK293T cells were either stably transduced with the ISRE-GF reporter constructs or transiently transfected with pRL-TK and ISG54-luciferase vectors along with the indicated plasmids. At 24 h after transient transfection, cells were treated overnight with universal IFN type I (1,000 U/mL) and luciferase activity was measured using the Dual-Luciferase Assay System (Promega) according to the manufacturer’s instructions. Firefly luciferase values were normalized to Renilla, and the fold induction was calculated as the ratio of IFN-stimulated versus unstimulated cells. For infection studies, hACE2-293T-ISRE-GF cells were infected with SARS-CoV-2 at the indicated MOI for 24 h. Subsequently, cells were stimulated with universal IFN type I (100 U/mL) for 16 h. Cells were lysed and luciferase activity was measured using the One-GLO Luciferase Assay System (Promega) according to the manufacturer’s instructions.

### Western Blot and Immunoprecipitation.

Vero E6 or hACE2-293T-ISRE-GF cells were infected with SARS-CoV-2 at the indicated MOI in viral growth media for 24 h. Subsequently, cells were either lysed directly or stimulated with universal IFN type I (1,000 U/mL) for 45 min or 16 h before lysis. SARS-CoV-2-infected cells were lysed in radio-immunoprecipitation assay (RIPA) buffer (Sigma-Aldrich) supplemented with 1% sodium dodecyl sulfate, cOmplete protease inhibitor mixture (Roche), and Halt phosphatase inhibitor mixture (Thermo Fisher Scientific). Lysates were boiled, sonicated three times at an output level of 2.5 W for 1 s, supplemented with 2× sample buffer (Bio-Rad Laboratories), and boiled again. For Western blot analysis, lysates were run on a 4 to 20% gradient gel and transferred onto polyvinylidene fluoride (PVDF) membranes (Bio-Rad Laboratories). Membranes were blocked in 5% nonfat dry milk-containing Tris-buffered saline with 0.1% Tween 20 detergent (TBS-T). Primary antibodies were used at dilutions of 1:1,000 and secondary horseradish peroxidase-conjugated antibodies were used at dilutions of 1:10,000 in 3% bovine serum albumin (BSA)-containing TBS-T. For coimmunoprecipitation, HEK293T cells were seeded in six-well format and transfected with the indicated plasmids or with an empty vector control. At 24 h after transfection, cells were lysed in RIPA buffer (Sigma-Aldrich) supplemented with cOmplete protease inhibitor mixture (Roche). Lysates were mixed in a 1:1 ratio as described and incubated overnight at 4 °C with anti-HA magnetic beads (Thermo Fisher Scientific). Beads were washed extensively and eluates were analyzed by Western blot as described above. Immunoprecipitation of Flag-Orf6 or endogenous Nup98 and Rae1 was performed as we previously reported (60).

### Confocal Microscopy Studies.

Vero E6, HEK293T, or hACE2-293T-ISRE-GF cells were seeded into 24-well glass bottom plates (MatTek) the day before infection or transfection. For infection studies, cells were infected with SARS-CoV-2 at the indicated MOI for 24 h, stimulated for 45 min with universal IFN type I (1,000 U/mL), and then fixed with 4% formaldehyde. For transient transfection studies, cells were either mock treated or treated with IFN-I (1,000 U/mL) 24 h after transfection and then fixed as described above. Cells were permeabilized with 0.2% Triton X-100 in phosphate-buffered saline (PBS), and blocked in PBS 0.1% Tween-20 with 3% BSA. Anti-dsRNA, anti-ACE2, anti-STAT2, and anti-pSTAT1 antibodies were used at dilution of 1:200; anti-HA, anti-T7, anti-FLAG, and anti-Myc antibodies were used at dilution of 1:500; the rabbit anti-SARS-CoV nucleoprotein was used at a 1:5,000 dilution, and the monoclonal mAb 1C7 at a 1:500 dilution. DAPI (4′,6-diamidino-2-phenylindole) and fluorophore-conjugated secondary antibodies were diluted 1:1,000. All antibodies were diluted in PBS 0.1% Tween-20 with 1% BSA. Samples were incubated with primary antibodies overnight at 4 °C, and secondary antibodies for 1 h at room temperature. Images were acquired as described elsewhere (61). Confocal laser scanning microscopy was performed with a Zeiss LSM880 confocal laser scanning microscope (Carl Zeiss Microimaging) fitted with a Plan Apochromat 63×/1.4 or 40×/1.4 oil objective. Images were analyzed with Fiji software (https://fiji.sc/), and the experiments were repeated at least three times.

### EMSA.

Orf6 (43–61) was synthesized with a FITC fluorophore at the N terminus. The Rae1•Nup98(157-213) complex was purified from insect cells as previously described (62). A total of 0.05 µM of FITC-Orf6 (43–61) was incubated with increasing concentrations of the Rae1•Nup98(157-213) complex in a buffer containing 10 mM Tris, pH 8.0, 100 mM NaCl, 0.5 mM Tris (2-carboxyethyl) phosphine, and 8% glycerol at 4 °C for 30 min. Samples were separated on a 5% native polyacrylamide gel electrophoresis gel that was prepared with 45 mM Tris base and 45 mM boric acid and prerun in the same buffer. After electrophoresis, FITC signals were visualized by a Typhoon FLA 9000 biomolecular imager (GE Healthcare).

### Single-Cell RNA-Seq.

For scRNA-Seq, Vero E6 cells were infected with SARS-CoV-2 (MOI 0.5) USA-WA1/2020 or mock infected for 8 h, and then were either left unstimulated or stimulated with IFN-I (100 IU/mL), IFN-II (10 ng/mL), or IFN-III (100 IU/mL) for 6 h. Cultures were dissociated to single-cell suspension, and independently labeled with MULTI-Seq barcode lipid-modified oligonucleotides (generously provided by Zev J. Gartner, University of California, San Francisco, CA) containing a sequence complementary to the 10× Genomics GEM 5′ capture sequence (63). Cells were pooled in equal proportions and processed for scRNA-Seq on the 10× Genomics Chromium Controller using 5′ Next GEM v1.1 reagents (10× Genomics, Inc) according to the manufacturer’s protocol. MULTI-Seq libraries were prepared as described (63). Gene expression and MULTI-Seq libraries were sequenced on an Illumina NextSEq 500 instrument to an approximate read depth of 82,000 reads/cell (gene expression) and 5,439 reads/cell (MULTI-Seq). Sequencing data were processed with CellRanger v3.1.0 (10× Genomics, Inc). Reads (gene expression libraries) were aligned and quantified to the African Green Monkey *Chlorocebus sabaeus* reference genome (ChlSab1.1, Ensembl v100.1) appended with SARS-CoV-2 genome reference (WuhCor1, NC_045512.2, modified to reflect the USA-WA1/2020 strain, MT246667.1) by CellRanger count. Reads from MULTI-Seq libraries were quantified against a barcode reference by CellRanger count using the “Antibody Capture” library setting. Gene x cell matrix (gene expression) and sample barcode x cell matrix (MULTI-Seq) were then analyzed with Seurat v3.1.5 (64). After an initial filter to remove cells with fewer than 300 UMIs and percent mitochondrial gene expression greater than 1%, data were demultiplexed with deMULTIplex (version 1.0.2) (63). Cells classified as doublets (by detection of two or more MULTI-Seq labels) or MULTI-Seq label negative were excluded from analysis. After filtering, 4,188 cells remained for analysis. Detailed methodology can be found in *SI Appendix*.

## Supplementary Material

Supplementary File

Supplementary File

Supplementary File

Supplementary File

Supplementary File

Supplementary File

Supplementary File

Supplementary File

Supplementary File

Supplementary File

Supplementary File

Supplementary File

## Data Availability

Single-cell RNA-Seq data have been deposited in the Gene Expression Omnibus (GEO) database, https://www.ncbi.nlm.nih.gov/geo (accession no. GSE159593).
