# Supplementary material for: SARS-CoV-2 Orf6 hijacks Nup98 to block STAT nuclear import and antagonize interferon signaling
Source: Proc Natl Acad Sci U S A. 2020 Oct 23;117(45):28344–54. doi: 10.1073/pnas.2016650117 (PMC7668094; doi:10.1073/pnas.2016650117)
Supplement: Supplementary File [file pnas.2016650117.sapp.pdf]

PNAS
www.pnas.org

Supplementary Information for

SARS-CoV-2 Orf6 hijacks Nup98 to block STAT nuclear import and antagonize interferon signaling

Lisa Miorin[1,2,#,*], Thomas Kehrer[1,2,#], Maria Teresa Sanchez-Aparicio[1,2,#], Ke Zhang[3] , Phillip Cohen[1], Roosheel S Patel[1], Anastasija Cupic[1,2], Tadashi Makio[4], Menghan Mei[5], Elena Moreno[1,2], Oded Danziger[1], Kris M White[1,2], Raveen Rathnasinghe[1,2], Melissa Uccellini[1,2], Shengyan Gao[3], Teresa Aydillo[1,2], Ignacio Mena[1,2], Xin Yin[6], Laura Martin-Sancho[6], Nevan J Krogan[1,7,8,9], Sumit K Chanda[6], Michael Schotsaert[1,2], Richard W Wozniak[4], Yi Ren[5], Brad R Rosenberg[1], Beatriz M A Fontoura[3], Adolfo García-Sastre[1,2,10,11,*]

[1]Department of Microbiology, Icahn School of Medicine at Mount Sinai, New York, NY, USA; [2]Global Health Emerging Pathogens Institute, Icahn School of Medicine at Mount Sinai, New York, NY, USA; [3]Department of Cell Biology, University of Texas Southwestern Medical Center, Dallas, Texas, USA; [4]Department of Cell Biology, University of Alberta, Edmonton, Alberta, Canada; [5]Department of Biochemistry, Vanderbilt University School of Medicine, Nashville, TN, USA; [6]Immunity and Pathogenesis Program, Infectious and Inflammatory Disease Center, Sanford Burnham Prebys Medical Discovery Institute, La Jolla, CA, USA; [7]Quantitative Biosciences Institute (QBI), University of California San Francisco, San Francisco, CA, USA; [8]J. David Gladstone Institutes, San Francisco, CA, USA; [9]Department of Cellular and Molecular Pharmacology, University of California San Francisco, San Francisco, CA, USA; [10]Department of Medicine, Division of Infectious Diseases, Icahn School of Medicine at Mount Sinai, New York, NY, USA; [11]Tisch Cancer Institute, Icahn School of Medicine at Mount Sinai, New York, NY, USA

[#]These authors contributed equally

Paste corresponding author name here
Email: adolfo.garcia-sastre@mssm.edu and lisa.miorin@mssm.edu

This PDF file includes:

Supplementary text
Figures S1 to S8
Legends for Movies S1 to S2
Legends for Datasets S1 to S9
SI References

Other supplementary materials for this manuscript include the following:

Movies S1 to S2
Datasets S1 to S9

www.pnas.org/cgi/doi/10.1073/pnas.2016650117

Supplementary Materials and Methods

Plasmids and transfection

The SARS-CoV-2 (USA-WA1/2020) and SARS-CoV (TOR2 strain) Orf6 coding sequence were cloned into pCAGGS mammalian expression vector that encodes a carboxyterminal HA-tag using NotI and KpnI restriction sites. Overlap-PCR was used to generate the $Orf6_{M58R}$ mutant by changing residue 58 from methionine (ATG) to arginine (CGG). Flag-tagged SARS-CoV2 Orf6 wildtype and $Orf6_{M58R}$ were cloned into the Xho I/Sma I sites of the pCI-neo-3XFlag vector. All expression vectors were confirmed by sequencing and are available upon request. The expression vectors for HA-NS5 ZKV (Uganda strain)(1), STAT1-GFP, STAT2-RFP(2), Myc-NUP98 (3), pRL-TK, and ISG54-Firefly luciferase plasmid have been previously described (4). pCMVTNT-T7-KPNA1 and pCMVTNT-T7-KPNA2 were a gift from Bryce Paschal (Addgene #26677, and #26678)(5). pEGFP-N1 Full length Importin beta was a gift from Patrizia Lavia (Addgene plasmid #106941)(6). All HEK293T and Vero E6 transfections were performed using TransIT-LT1 (Mirus Bio) according to the manufacturer's instructions.

Antibodies and cytokines.

Primary antibodies used in this study were: anti-HA-HRP (Cell Signaling, 6E2), anti-HA (Cell Signaling, C29F4), anti-myc (Cell Signaling 9B11), anti-T7 (Cell Signaling, D9E1X), M2 anti-Flag (Sigma Aldrich, F1804), anti-tubulin (Sigma Aldrich, T8328), anti GAPDH (Santa Cruz, sc-365062), anti-total STAT1 (Santa Cruz, sc-417), anti-total STAT2 (Santa Cruz, sc-476), anti-phospho-STAT1 Y701 (Cell Signaling, 58D6), anti-phospho-STAT2 Y690 (Cell Signaling, D3P2P), monoclonal anti-SARS-CoV nucleoprotein (mAb 1C7, gift from Thomas Moran), rabbit polyclonal anti-SARS nucleoprotein (produced in house), anti-GFP (Santa Cruz, sc-8334), J2 anti-dsRNA (Scicons, 10010500), IFIT3 (Proteintech, 15201-1-AP), anti-Nup98 (7), anti Rae1 (ThermoFisher, PA5-93166), anti-ACE2 (R&D systems, cat#AF933). Secondary antibodies were: Anti-mouse-HRP (Kwik, Cat# 1005), anti-rabbit-HRP (Kwik, Cat# 1006), anti-mouse-AlexaFluor488 (Invitrogen, A11029), anti-mouse-AlexaFluor594 (Invitrogen, A21203), anti-mouse-AlexaFluor647 (Invitrogen, A21235), anti-rabbit-AlexaFluor594 (Invitrogen, A21207), anti-rabbit-AlexaFluor647 (Invitrogen, A31573). Cytokines used in this study were: Universal Type I Interferon Alpha (PBL Assay Science, cat# 11200-2), human interferon gamma (PBL Assay Science, cat# 11500-2), human interleukin 29/interferon lambda 1 (PBL Assay Science, cat# 11725-1).

Stimulated emission depletion (STED) microscopy imaging.

HEK293T cells expressing Flag-Orf6 plasmid were fixed with 3.7% formaldehyde in PBS for 10 min, permeabilized with 0.1% Triton X-100 in PBS for 10 min, washed with PBS-T (PBS + 0.1% Tween 20) and blocked with PBS-T + 1% BSA for 20 min. All steps were performed at RT. The samples were incubated with a primary antibody solution containing a mouse anti-Flag antibody (1:1000, Sigma) and either rabbit anti-Nup98 antibodies (1:100) or anti-Nup358 antibodies (1:1000) in PBS-T + 1% BSA for 1 h at RT. The samples were further incubated with a secondary antibody solution containing Alexa488 anti-mouse IgG antibodies (1:1000, Invitrogen) and Alexa555 anti-rabbit IgG antibodies (1:1000, Invitrogen), or Alexa594 anti-mouse IgG antibodies (1:1000, Invitrogen) and Atto647N anti-rabbit IgG antibodies (1:1000, Sigma) in PBS-T + 1% BSA for 1 h at RT, and then washed with PBS-T. The coverslips containing the cell samples were mounted on DAPI-fluoromountG (SouthernBiotech) or Mowiol (polyvinyl alcohol) mounting media. STED imaging was performed on a Leica SP8 STED system equipped with a STED-dedicated HC PL APO CS2 100x/1.40 OIL objective (Leica). Dual-color STED experiments were performed by sequential imaging of Alexa594 (excited by 592 nm laser) and Atto647N (excited by 640 nm laser) channels with a 775 nm pulsed depletion laser. The images were sampled at 28nm per pixel with

a pixel dwelling at 200 Hz. The images were further processed with ImageJ (NIH). Series of z-stack images used for movies were acquired using a DeltaVision Elite imaging system (GE Healthcare) equipped with a PlanApo N 60x/1.42 NA oil objective (Olympus) at 0.24 µm intervals along the z axis. Images were deconvolved with the deconvolution module of the SoftWoRx software (GE Healthcare) under a “conservative” setting. Deconvolved images were further processed (crop, normalization) with ImageJ (NIH). For the analysis of the distance distribution between Flag-Orf6 and the NPC the regions of images representing flat surface areas of the nuclear envelope (the bottom of the nucleus) were manually extracted and local maxima of the Flag-Orf6 and Nup358 signals within the regions were listed as spot coordinates using ImageJ. The distance from each Flag-Orf6/Flag-Orf6M58R spot to the nearest NPC (Nup358) was calculated and summarized.

Single-cell RNAseq

Classification of SARS-CoV-2 Infection Status

Cells were classified as SARS-CoV-2 infected, bystander, mock or undetermined based on gene-specific expression thresholds for all SARS-CoV-2 subgenomic RNAs (sgmRNAs). Thresholds were derived on a per gene basis by examining sgmRNA expression in infected and mock infected samples and defining limits that effectively separated cells with high and low signal for each sgmRNA (Figure S3). A cell is classified as “infected” if it expresses all 10 sgmRNAs above thresholds (normalized RNA expression values of S ≥ 2.2, ORF3a ≥ 2.2, E ≥ 1.5, M ≥ 4, ORF6 ≥ 2.8, ORF7a ≥ 4, ORF7b ≥ 3, ORF8 ≥ 4, N ≥ 6.5, and ORF10 ≥ 4.2). A cell is classified as “bystander” if it is present in a SARS-CoV-2 infected culture and has normalized RNA expression values for all 10 sgmRNAs below the following cutoffs: S ≤ 0.8, ORF3a ≤ 1, E ≤ 0.7, M ≤ 1.5, ORF6 ≤ 1.2, ORF7a ≤ 1.5, ORF7b ≤ 1, ORF8 ≤ 1.2, N ≤ 3.5, and ORF10 ≤ 1.5. Cells from infected cultures that did not satisfy these conditions were classified as “undetermined”. Any cells from mock infected cultures classified as "infected" by sgmRNA counts were presumed to be doublets and removed from downstream analyses. All remaining cells from mock-infected samples were classified as “mock.”

Data visualization

Viral gene counts were excluded for downstream analyses. Per cell transcript counts (UMIs) were normalized with SCTransform implemented in the Seurat package (8) with cell cycle (as determined by Seurat’s CellCycleScoring function) and percent mitochondrial gene expression included for regression. Principal components analysis was performed on the top 3,000 most variable genes and the first twenty components were used for Uniform Manifold Approximation and Projection (UMAP) plots in Figure 4A.

Differential gene expression analysis

Differential gene expression (DGE) analyses were conducted using edgeR v3.30.3 (9, 10), with additional modifications for scRNA-Seq data (11). Gene x cell count matrices were extracted from Seurat objects with infection and stimulation information as metadata. Viral genes were excluded from all differential gene expression analyses and cell cycle scores were calculated using Seurat’s CellCycleScoring function. For all analyses, genes expressed (i.e. greater than or equal to 1 UMI) in less than 10% of cells for at least one group were excluded from differential gene expression testing. To identify IFN-I, IFN-II, and IFN-III ISGs in Vero E6 cells, differential expression analysis was conducting comparing mock cells stimulated with each IFN to corresponding unstimulated mock cell controls. edgeR linear models included factors for cell cycle score, cellular gene detection rate, and stimulus condition. For each IFN, significantly upregulated genes (adjusted p value < 0.05 and $\log_2 FC > 1$) in the stimulated group were designated ISGs. Additional differential gene expression tests were conducted comparing infection status and/or IFN stimulation. In these contrasts, to mitigate the large differences in host gene expression for mock and bystander cells relative to infected cells (Figure 4B), transcript counts from mock and bystander cells were downsampled as follows. Within each stimulation group (IFN-I, IFN-II, IFN-III, or unstimulated), transcript counts for each cell were randomly downsampled to the median transcript counts/cell of the infected cells group. All cells with counts below this value were excluded from differential

expression analyses. The resulting counts matrix was used in edgeR differential expression testing. Gene expression linear models included factors for cellular gene detection rate, cell cycle scores, stimulus condition and infection status (classified as described above). Contrasts included testing for differences between infection conditions (i.e. within a given IFN stimulation condition) and testing for differences in the IFN response (IFN stimulated vs. IFN unstimulated) across infection status (infected vs. bystander, infected vs. mock, bystander vs. mock). Significant differential induction was defined as adjusted p value < 0.05 and moderated $\log_2$ fold-change difference > 0.58.

Gene ontology term enrichment testing

Gene ontology (GO) term enrichment analyses were conducted with the goana function (edgeR) for Biological Process terms (BP, Homo sapiens). Gene lists were converted to Homo sapiens ENTREZ identifiers by matching gene symbols. Background gene list included all expressed (i.e. greater than or equal to 1 UMI in greater than 10% of cells for at least one group) ENTREZ genes evaluated by differential gene expression testing. Enrichment p values were adjusted for multiple testing by the method of Benjamini and Hochberg (p.adjust function, stats v4.0.2). GO BP terms with adjusted p value < 5x10-6 are reported in Dataset S9.

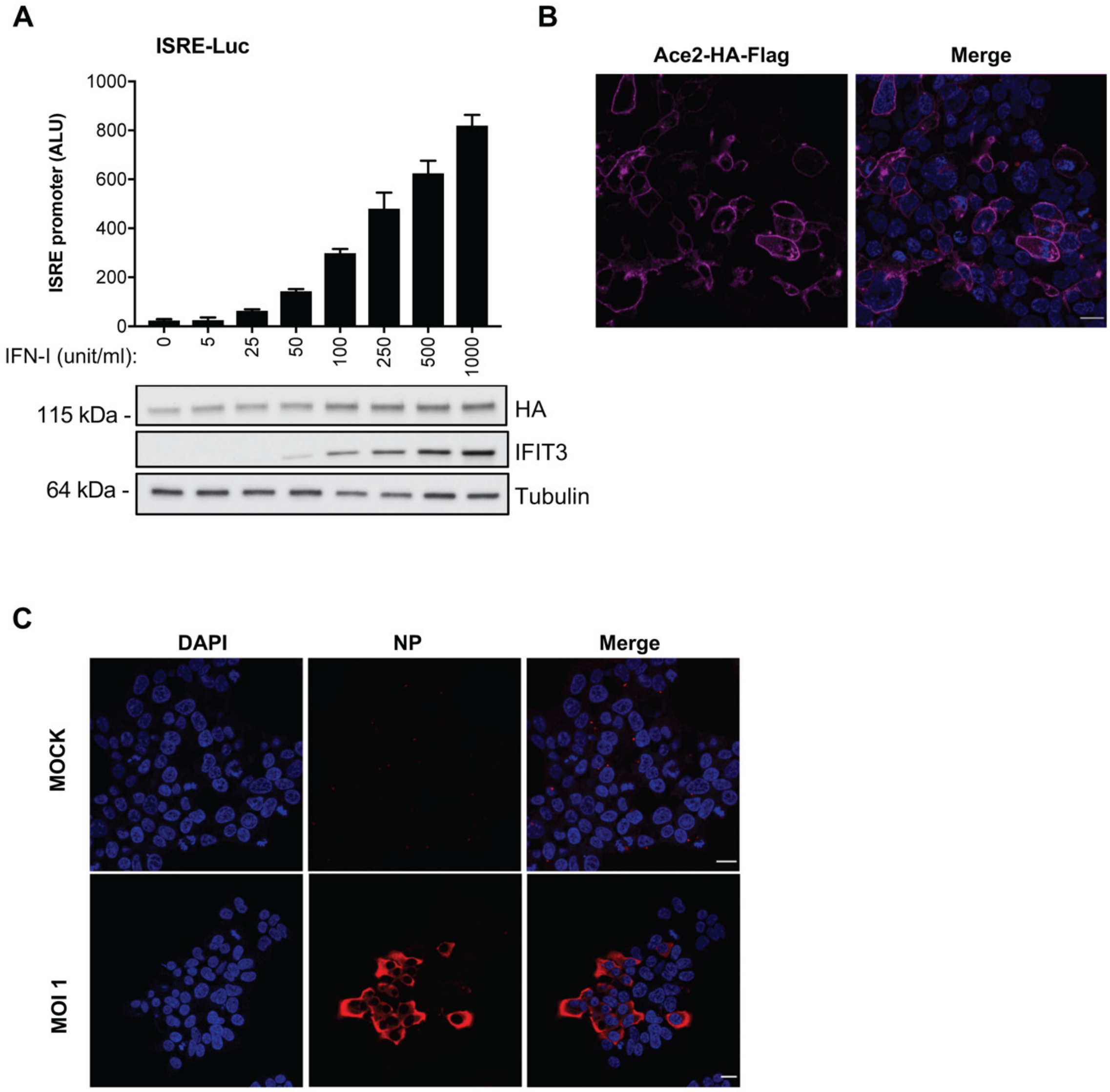

Fig. S1. Characterization of the hACE2-293T-ISRE-GF reporter cell line. (A) hACE2-293T-ISRE-GF cells were treated with increasing amounts of IFN-I as indicated in the figure. 24 hr after treatment, cells were lysed and luciferase activity was measured. Data are represented as average +/- SD (n=3). ALU, absolute light unit. Cell lysates from the reporter assay were analyzed by Western blot to show expression of hACE2 (HA), and the interferon stimulated gene IFIT3. Tubulin was used as loading control. **(B)** Confocal microscopy images of hACE2-293T-ISRE-GF cells stained with an anti-Flag antibody showing surface expression of hACE2-HA-Flag. Scale bars = 20 µm. **(C)** Confocal microscopy images of hACE2-293T-ISRE-GF cells infected with SAR-CoV-2 for 24h at MOI 1. Cells were stained for NP with DAPI counterstain. Scale bars = 20 µm.

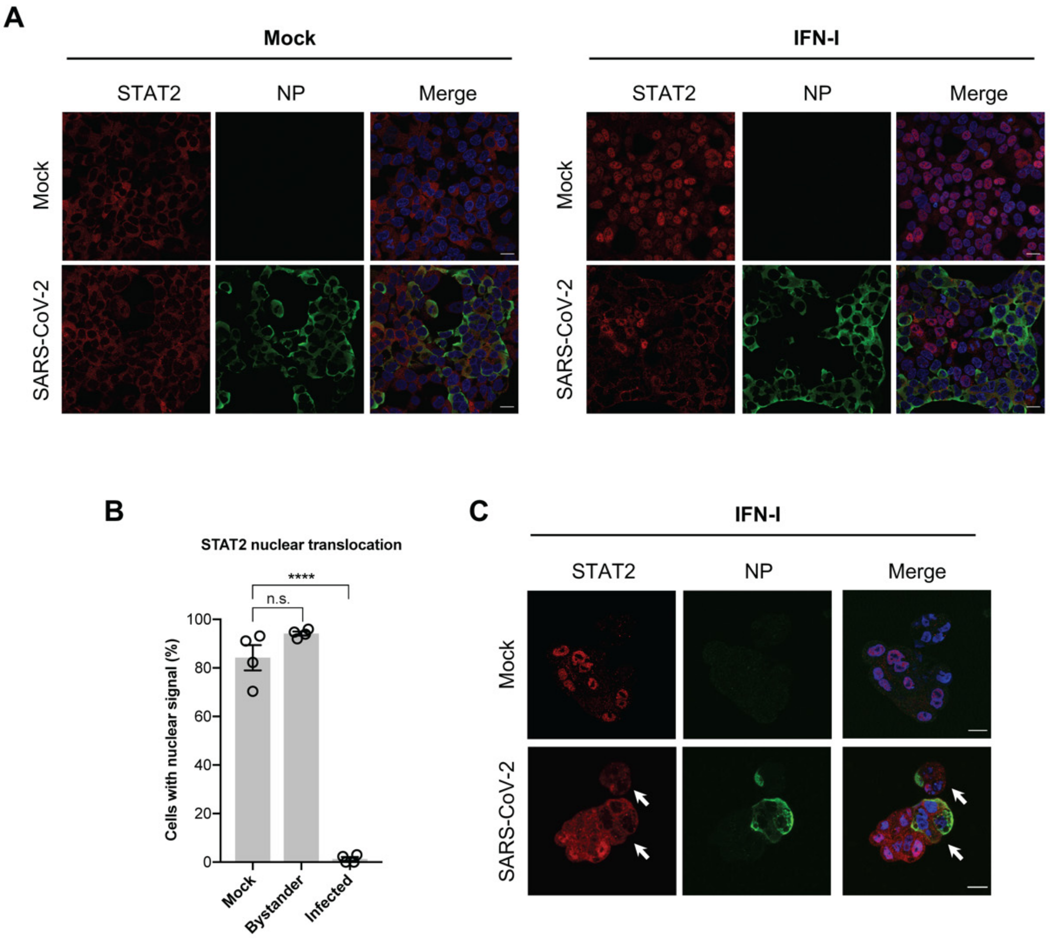

Fig. S2. SARS-CoV-2 infection impairs STAT2 nuclear translocation in hACE2-293T and Calu3 cells. (A) hACE2-293T-ISRE-GF cells were mock infected or infected with SARS-CoV-2 at an MOI of 2. At 24 hr post infection, cell were either mock treated or treated with IFN-I (1000 U/ml) for 45 min. The subcellular localization of STAT2 was analyzed by confocal microscopy. Nuclei were stained with DAPI. Scale bars = 20 μm. **(B)** Quantification of STAT2 nuclear translocation in uninfected, bystander and infected cells from 4 fields of view ($n \geq 300$). Data are shown as average +/- SEM. Significance was determined by unpaired two-tailed t-test. $p > 0.05$ = n.s. ; $p < 0.0001$ = ****. **(C)** Calu3 cells were mock infected or infected with SARS-CoV-2 at an MOI of 2 and treated as described in (A).

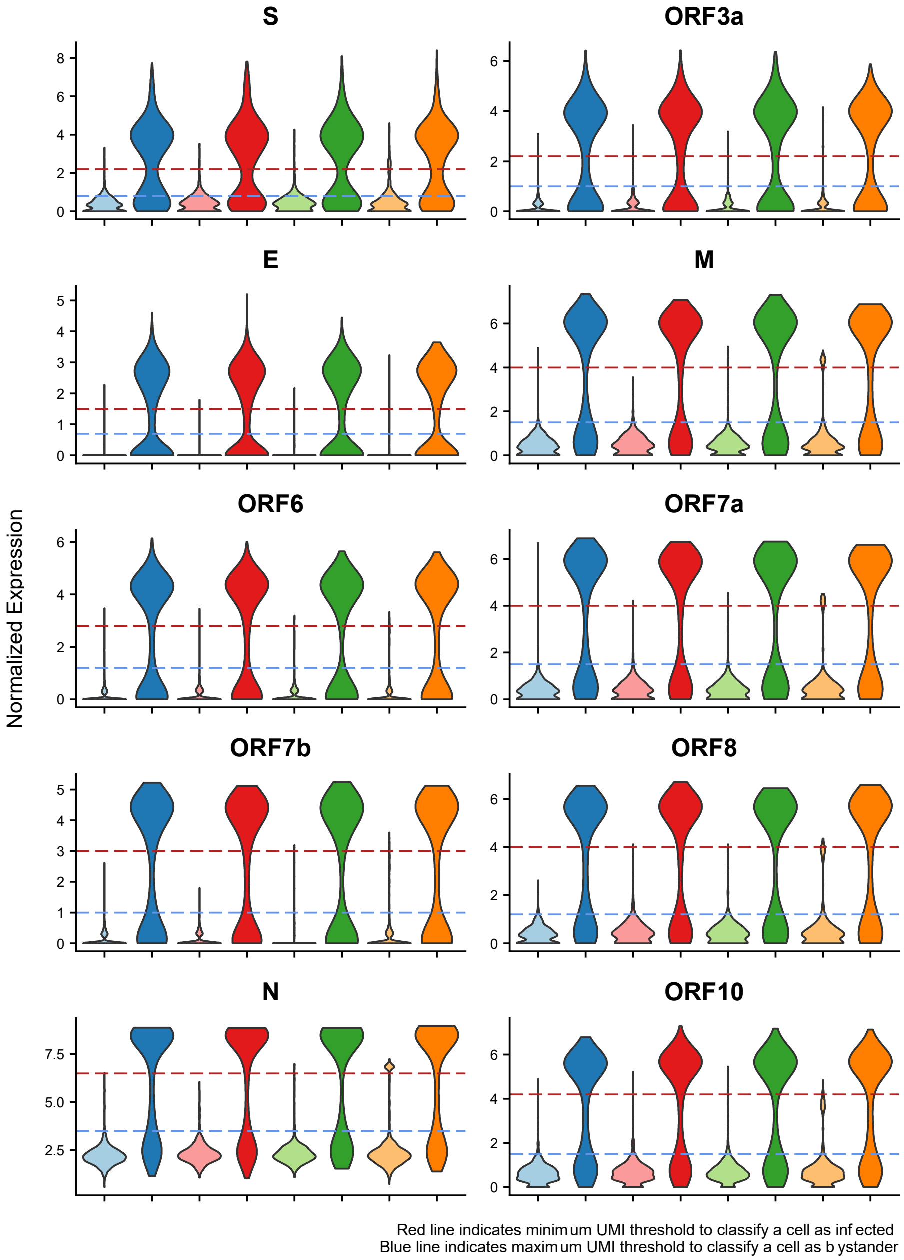

Fig. S3. Expression of SARS-CoV-2 sgmRNAs. Expression of SARS-CoV-2 sgmRNAs. Classification thresholds were set on a per-gene basis to distinguish cells with high viral sgmRNA expression from cells with low sgmRNA expression. Red dashed lines indicate the lower expression threshold for a cell to be classified as infected. Blue dashed lines indicate the upper expression threshold for a cell to be classified as bystander or mock. Cells with expression between these bounds or without all 10 sgmRNAs classified in the same manner were classified as undetermined.

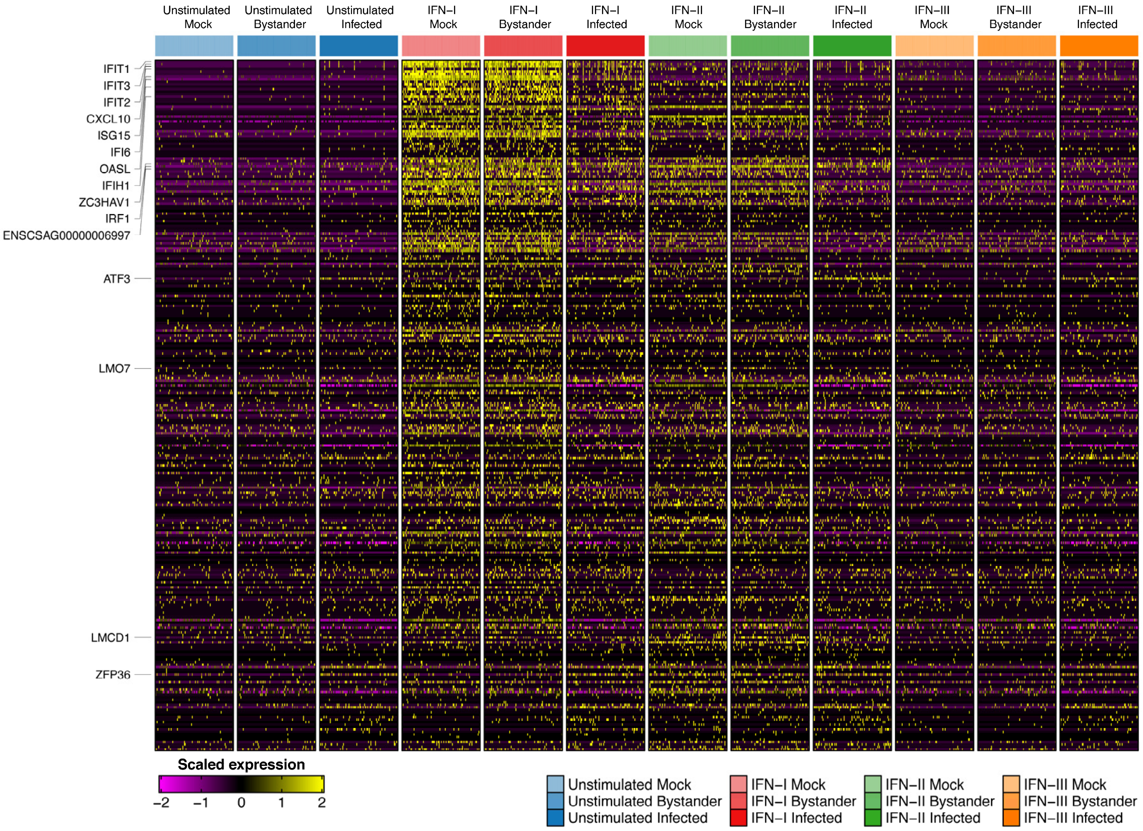

Fig. S4. ISG induction heatmap. ISG induction upon treatment of infected, bystander, and mock cells with IFN-I, IFN-II, or IFN-III. Gene symbols indicate ISGs with significant differential induction (i.e. IFN stimulated vs unstimulated between different infection conditions) in IFN-I or IFN-II stimulation comparisons (Figure 4D). Comprehensive ISG lists for IFN-I, IFN-II, and IFN-III are presented in Dataset S1.

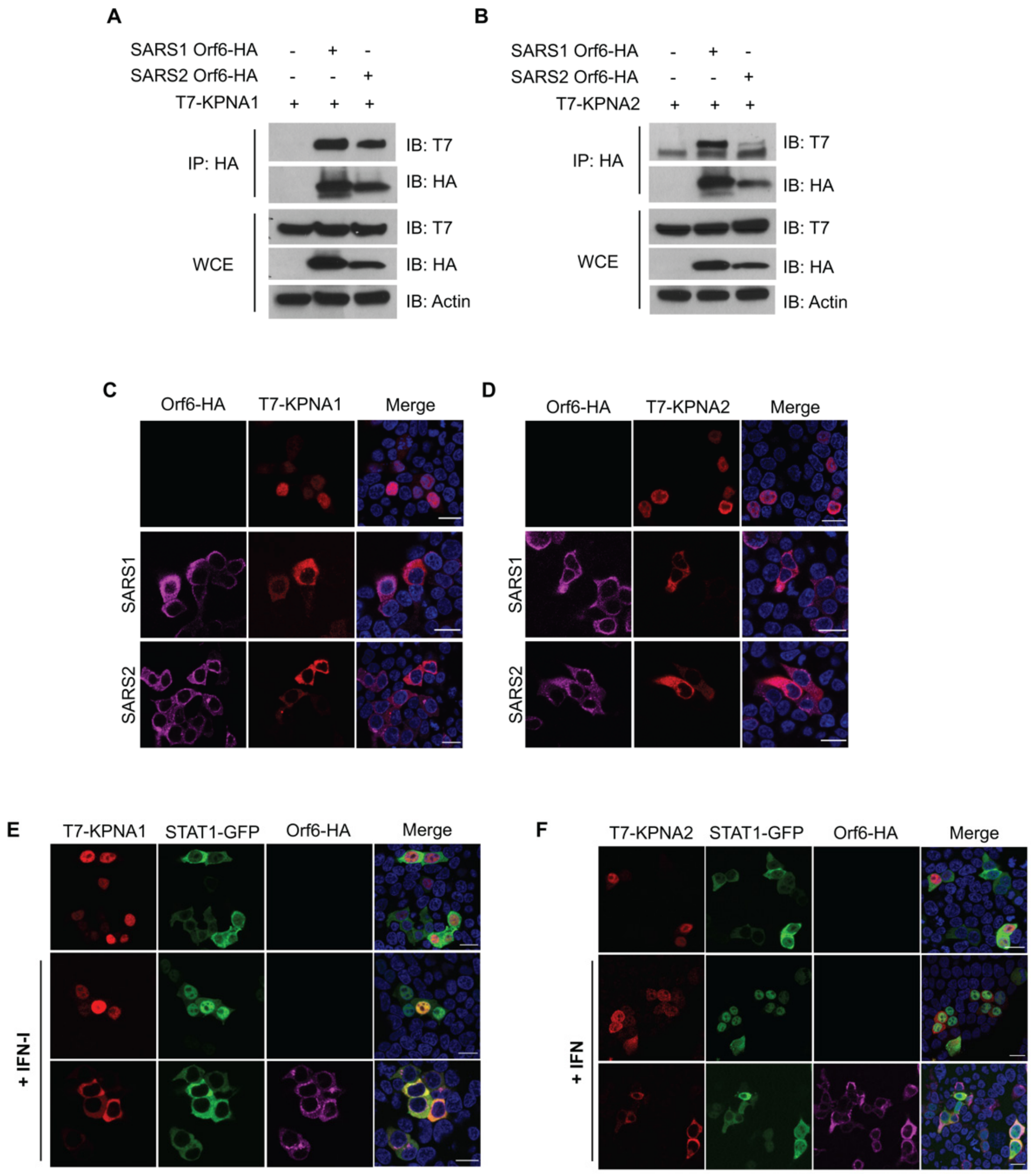

Fig. S5. Orf6 interacts with KPNA1 and KPNA2 in the cytoplasm. (**A-B)** HEK293T cells were transfected for 24 h with the indicated expression vectors. Cell lysates were subjected to immunoprecipitation (IP) using anti-HA antibody and followed by immunoblot with the antibodies indicated in the figure. **(C-D)** Confocal microscopy images of HEK293T cells ectopically expressing SARS-CoV (SARS1) or SARS-CoV-2 (SARS2) Orf6-HA along with either T7-KPNA1 (C), T7-KPNA2 (D). Twenty-four hours post-transfection, cells were fixed and immunostained with the indicated antibodies. **(E-F)** Confocal microscopy images of HEK293T cells transfected with SARS-CoV-2 Orf6-HA along with T7-KPNA1 (E), T7-KPNA2 (F). At 24 h post-transfection, cells were mock stimulated or stimulated for 45 min with IFN-I (1000 U/ml) to monitor STAT1 and STAT2 subcellular localization. Scale bars = 20 μm.

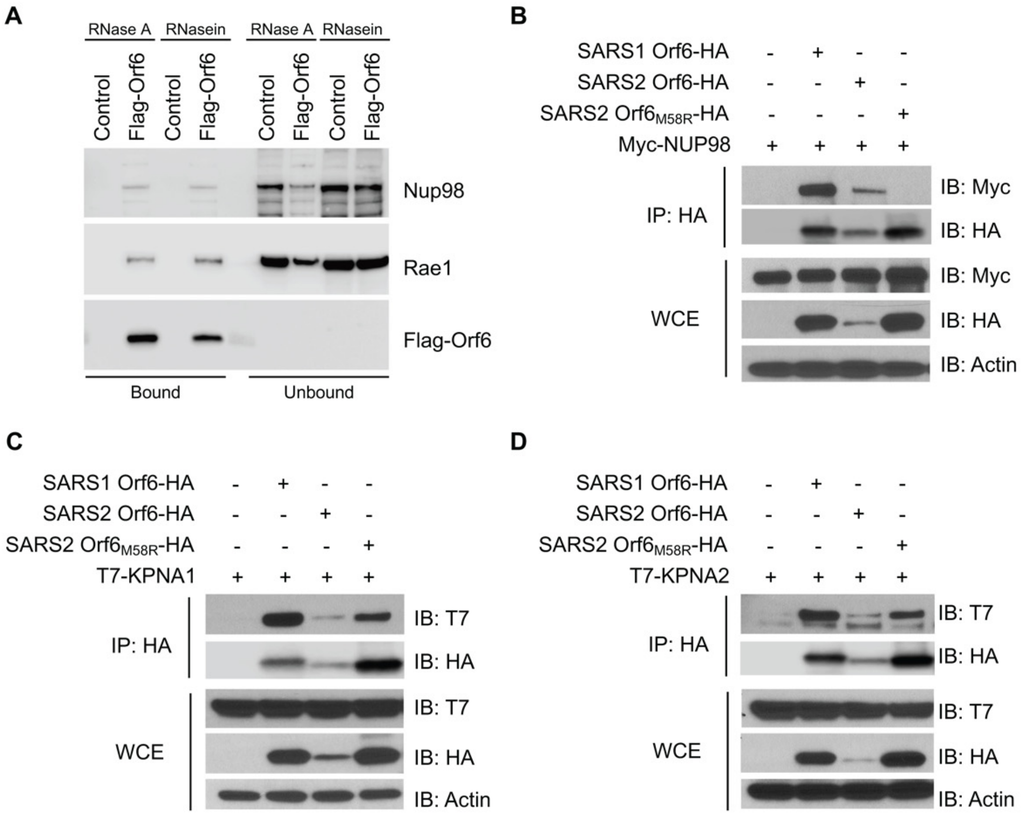

Fig. S6. SARS-CoV and SARS-CoV-2 Orf6 interact with Nup98, KPNA1 and KPNA2. (A) HEK293T cells were transfected for 24h with Flag-Orf6, or vector control. Cell lysates were subjected to immunoprecipitation (IP) using anti-Flag antibody and followed by immunoblot (IB) with antibodies against the depicted proteins. **(B-D)** HEK293T cells were transfected for 24 hr with the indicated expression vectors. Cell lysates were subjected to immunoprecipitation (IP) using anti-HA antibody and followed by immunoblot with the antibodies indicated in the figure.

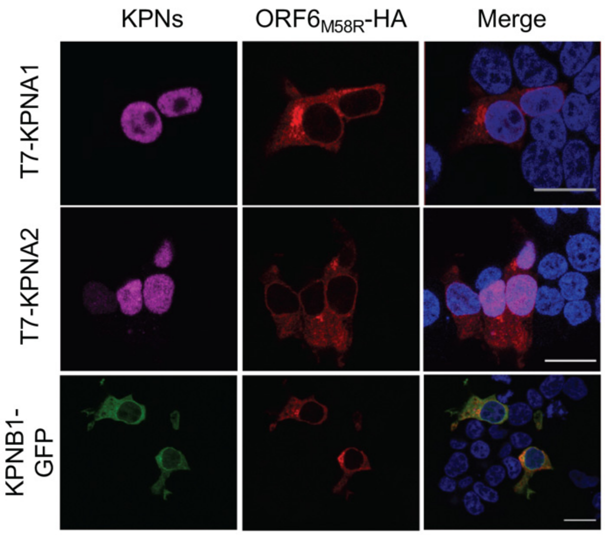

Fig. S7. SARS-CoV-2 Orf6$_{M58R}$ does not retain KPNA1 and KPNA2 into the cytoplasm. Confocal microscopy images of HEK293T cells transfected with Orf6$_{M58R}$-HA along with T7-KPNA1, T7-KPNA2, or KPNB1-GFP. At 24 h post-transfection, cells were fixed and immunostained with the indicated antibodies. Scale bars = 20 μm.

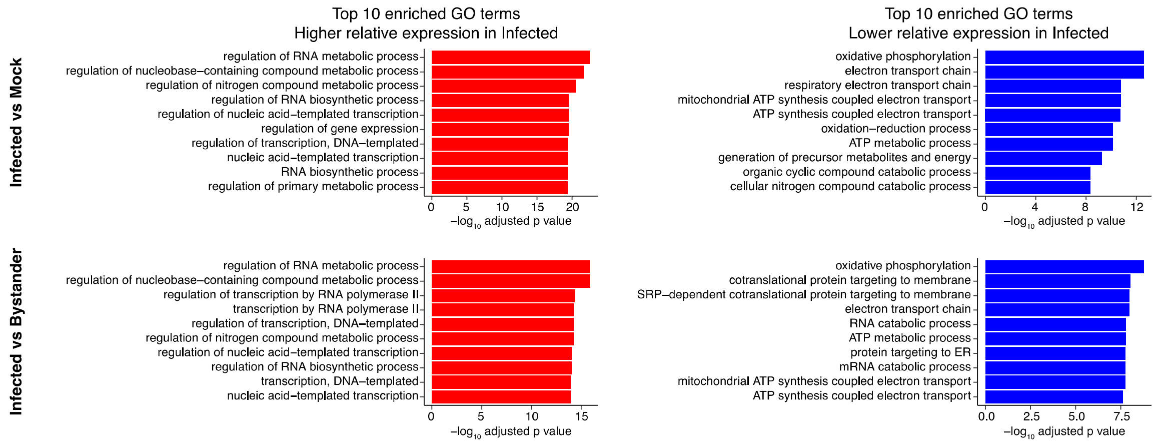

Fig. S8. Gene Ontology (GO) term enrichment for differentially expressed genes. Top 10 (ranked by adjusted p-value) GO terms (Biological Process) for differentially expressed genes in the indicated contrasts. All contrasts were performed on unstimulated (i.e. no IFN) conditions. For bystander vs. mock contrasts, no genes met significance thresholds (log2 fold-change > 1, FDR 0.05) for inclusion in GO term enrichment analyses. Complete lists of all significant (adjusted p value < 5 x 10-6) enriched GO terms are presented in DatasetS9.

Movie S1 (separate file). Colocalization of Flag-Orf6 with the NPC protein Nup98. HEK293T cells ectopically expressing Flag-Orf6 were analyzed by indirect immunofluorescence using anti-Flag (green) and anti-Nup98 (red) antibodies. Series of z-stack images obtained using a DeltaVision microscope are shown as movies. Merged images are shown in the left most panel.

Movie S2 (separate file). Colocalization of Flag-Orf6 with the NPC protein Nup358. HEK293T cells ectopically expressing Flag-Orf6 were analyzed by indirect immunofluorescence using anti-Flag (green) and anti-Nup358 (red) antibodies as described in Supplementary video 1 legend.

Dataset S1 (separate file). IFN-I, IFN-II, IFN-III ISGs identified by differential expression testing (logFC > 1 and adjusted p value < 0.05).

Dataset S2 (separate file). Differential gene expression analysis comparing infected, bystander, and mock cells from untreated conditions.

Dataset S3 (separate file). Differential gene expression analysis comparing infected, bystander, and mock cells from IFN-I treated conditions.

Dataset S4 (separate file). Differential gene expression analysis comparing infected, bystander, and mock cells from IFN-II treated conditions.

Dataset S5 (separate file). Differential gene expression analysis comparing infected, bystander, and mock cells from IFN-III treated conditions.

Dataset S6 (separate file). Differential gene expression analysis comparing IFN-I stimulated vs unstimulated across infected, bystander, and mock cells.

Dataset S7 (separate file). Differential gene expression analysis comparing IFN-II stimulated vs unstimulated across infected, bystander, and mock cells.

Dataset S8. (separate file). Differential gene expression analysis comparing IFN-III stimulated vs unstimulated across infected, bystander, and mock cells.

Dataset S9. (separate file). Gene ontology (GO) term enrichment for differentially expressed genes.

SI References

1. A. Grant *et al.*, Zika Virus Targets Human STAT2 to Inhibit Type I Interferon Signaling. *Cell Host Microbe* **19**, 882-890 (2016).
2. J. Ashour *et al.*, Mouse STAT2 restricts early dengue virus replication. *Cell Host Microbe* **8**, 410-421 (2010).
3. B. M. Fontoura, G. Blobel, M. J. Matunis, A conserved biogenesis pathway for nucleoporins: proteolytic processing of a 186-kilodalton precursor generates Nup98 and the novel nucleoporin, Nup96. *J Cell Biol* **144**, 1097-1112 (1999).
4. G. A. Versteeg *et al.*, The E3-ligase TRIM family of proteins regulates signaling pathways triggered by innate immune pattern-recognition receptors. *Immunity* **38**, 384-398 (2013).
5. J. B. Kelley, A. M. Talley, A. Spencer, D. Gioeli, B. M. Paschal, Karyopherin alpha7 (KPNA7), a divergent member of the importin alpha family of nuclear import receptors. *BMC Cell Biol* **11**, 63 (2010).
6. M. Ciciarello *et al.*, Importin beta is transported to spindle poles during mitosis and regulates Ran-dependent spindle assembly factors in mammalian cells. *J Cell Sci* **117**, 6511-6522 (2004).

7. A. Radu, M. S. Moore, G. Blobel, The peptide repeat domain of nucleoporin Nup98 functions as a docking site in transport across the nuclear pore complex. *Cell* **81**, 215-222 (1995).
8. C. S. McGinnis *et al.*, MULTI-seq: sample multiplexing for single-cell RNA sequencing using lipid-tagged indices. *Nat Methods* **16**, 619-626 (2019).
9. D. J. McCarthy, Y. Chen, G. K. Smyth, Differential expression analysis of multifactor RNA-Seq experiments with respect to biological variation. *Nucleic Acids Res* **40**, 4288-4297 (2012).
10. M. D. Robinson, D. J. McCarthy, G. K. Smyth, edgeR: a Bioconductor package for differential expression analysis of digital gene expression data. *Bioinformatics* **26**, 139-140 (2010).
11. C. Soneson, M. D. Robinson, Bias, robustness and scalability in single-cell differential expression analysis. *Nat Methods* **15**, 255-261 (2018).
